# How to be SSB-free: Assessing the attitudes and readiness for a sugar sweetened beverage-free healthcare center in the Bronx, NY

**DOI:** 10.1371/journal.pone.0215127

**Published:** 2019-05-15

**Authors:** P. Christopher Palmedo, Lauren M. Gordon

**Affiliations:** Department of Community Health and Social Sciences, City University of New York School of Public Health and Health Policy, New York, NY, United States of America; Auburn University, UNITED STATES

## Abstract

In recent years, communities and institutions have sought new interventions intended to reduce sugar sweetened beverage (SSB) consumption among children. Among these interventions are “SSB-free zones,” where such beverages are not permitted to be consumed on the premises. Insufficient knowledge still exists, however, about the readiness for such restrictive SSB policies within health care institutions. Understanding attitudes toward SSB consumption among adults is necessary to guide an institution-wide policy, where staff and patients serve as role models for parents and their children. We conducted focus groups with health center patients and staff to determine perceptions surrounding health and SSB consumption and to better understand the support and readiness (or lack thereof) for an SSB-free zone intervention prior to its implementation. We found that contextual practices present challenges to breaking personal consumption habits, even if beverages are banned from the worksite. Nevertheless, participants expressed support for SSB-free zones, and recommended more education about the harmful effects of soda and energy drink consumption to help improve acceptability for the policy. We conclude that policies restricting onsite SSB consumption may be more effective when combined with educational information and expressions of understanding that this specific behavior change can be difficult.

## Introduction

Sugar-sweetened beverages (SSBs), which include soda, energy drinks and sweetened juices, are recognized as significant contributors to numerous adverse health conditions in the United States, particularly among children [1–5]. SSB consumption is now recognized as a predictor of obesity, hypertension, diabetes, heart disease, and cancer [6–8]. Sugar-sweetened beverage consumption contributes up to 46% of the added sugars in the U.S. diet,[9] and is becoming an increasing concern throughout the world [10]. African American and Hispanic Americans, disproportionately represented in the American obesity epidemic,[11] also consume disproportionally more SSBs than non-Hispanic whites[12]. Health-related organizations are increasingly endorsing SSB-free zones to address the harms caused by these products. In 2017, the American Medical Association House of Delegates called for new strategies to reduce SSB consumption, including restricting access in hospitals, schools, and other community-based settings[13].

In 2015, pediatricians and primary care providers at Union Community Health Center, a not-for-profit federally qualified health center in the Bronx, NY, initiated an institution-wide SSB-free policy due to increasing concern about obesity and diabetes among their young patients. In New York City, daily consumption of SSBs is higher in low-income neighborhoods,[14] and the Bronx has been found to have the highest rate of SSB consumption among New York boroughs, with 42.1% of its residents reported to drink at least one sweetened beverage per day in 2010[15]. Union was the first health center in New York State to introduce an SSB-free zone to address these health concerns[16]. and although the target audience for the SSB-free policy was parents and their children, health center leadership decided to create an comprehensive institution-wide policy to avoid the stigma and shame of singling out one targeted subpopulation group.

Previous research studies on tobacco and alcohol inform us that existing social practices can influence and normalize unhealthy behaviors[17]. For example, a workplace smoking ban may be ineffective if it causes resentment and lost productivity among workers, and if the unhealthy behavior is ultimately transferred to different locations (e.g. sidewalks and local parks),[18] instead of being eliminating altogether. Multilevel frameworks on change readiness and theories from social marketing have provided insights into designing tobacco reduction campaigns, which can help inform SSB-control strategies. Understanding the context in which social practices occur helps to better understand health behaviors and to inform targeted interventions to address those behaviors.

In this article, we discuss our qualitative study and its implications for designing an SSB-free zone in a community-based institution. The primary aim of our qualitative research was to understand the social practices, motivations and emotions behind SSB consumption, and to assess staff and patient attitudes about the imposition of the SSB-free zone before implementation. Our fundamental research question was to determine what could be done to ensure buy-in of an SSB-free policy at a community center.

## Methods

Approach: The study used focus group interviews with 30-second video clips at the end of each focus group to explore belief patterns and message framing of sugar-sweetened beverages among patients and employees of the community health clinic. The research team determined that focus groups would be an effective method for data collection given this technique’s utility in qualitative research to capture rich data about various perspectives on a given issue and in efficiently achieving data saturation.

Informed by the literature on social practice theory, multilevel frameworks on change readiness and social marketing, [19] we developed an interview guide consisting of five semi-structured questions centered on belief patterns and message framing of sugar-sweetened beverages (see Table 1: Focus group questions). After the question and answer portion of the focus groups, participants were shown four 30-second video clips to garner feedback on four messaging subthemes. One video, from the New York Department of Health and Mental Hygiene (NYC DOHMH), explained how sugary drinks contain more sugar than most people realize. Another, also from the NYC DOHMH, emphasized the weight gain that may result from consuming sugary drinks.[20] A third contained a news clip discussing targeted soda marketing to African-American and Latino communities in low-income neighborhoods. The fourth clip consisted of a news segment discussing sugar’s potentially detrimental effect on brain development.

**Table 1:** Focus group questions.

Focus Group Questions	Theme	Construct
What do you think about health?” What does health mean to you?” “Do you think it’s important to be healthy?”	Health	Social Practice Theory
What is healthy or unhealthy about the building and facilities here at Union Community Health Center?”	Healthy Environments	Social Practice Theory
What do you think about sugary drinks such as soda?” “Do you drink soda?”	Sugar-sweetened beverages (SSBs)	Social Practice Theory
What do you think about a soda-free zone at Union Community Health Center?	SSB-free zones	Social Marketing
What messages or strategies would better help you and others here at Union Community Health Center reduce consumption of SSBs?	Effective messaging and framing	Social Marketing

### Inclusion criteria

Patients, parents of patient, and employees at Union Community Health Center, between 18–65 years old were eligible to participate (n = 23).

### Participants and recruitment

Participants were recruited through health center email correspondence, flyers, and through face-to-face invitations. Participants were told they would be part of a focus group discussing their opinions about health and healthy lifestyles. Given the specific population criteria, convenience and snowball sampling techniques were deemed appropriate for recruitment.[21] Gift cards ($20) and meals were provided to participants as an incentive for their participation.

The goal was to recruit between 20 and 40 participants to achieve saturation and content validity.[21] We conducted four focus groups, two for patients and two for staff. Three of the four focus groups were conducted in English and one was conducted in Spanish to accommodate Spanish-speaking patients with limited English proficiency. The focus groups took place in June 2015, and were audio-recorded and professionally transcribed.

The sample included 13 females and 10 males who self-reported as African-American, African, Guyanese, Hispanic, South Asian Indian and White.

### Transcription, analysis and themes

A grounded theory-based coding matrix was used to identify the emergent themes from the interviews. The interviews were transcribed and entered into NVivo 10 software. A content analysis approach helped us better understand attitudes and beliefs about individual consumption and institution-level SSB policy.

Two researchers performed coding to increase inter-rater reliability of the data. The researchers discussed themes and subthemes and refined the coding if a disagreement occurred.

The ‘health’ theme had three subthemes: healthy behaviors, benefits of health, and health conditions. The ‘healthy environments’ theme encompassed participants’ thoughts about healthy environments and if Union was considered a healthy or an unhealthy environment. The subtheme here was environments that encourage or discourage health. The theme ‘sugary drinks’ encompassed participants’ thoughts about SSBs. The subthemes that emerged were usage and patterns, activities performed while drinking soda, emotional state while drinking soda, preferred brands and tastes, the influence of family and friends, and reactions to the SSB-free zone policy at Union.

## Results

Focus group participants’ responses to each of the themes and subthemes are indicated in Table 2: Summary of participant responses by theme from the focus groups. For the video portion of focus group, the goal was to explore participants’ reactions to different message framing about reasons to limit consumption of sugary beverages. The research also explored broad counter marketing messages against soda consumption through videos. Our study discovered limited understanding of the health effects of SSBs among participants in the patient focus groups; a concern recognized by the Food and Drug Administration in the food labeling requirements introduced in 2016[22].

**Table 2:** Summary of participant responses by theme from the focus groups.

Emerging Themes	Emerging Subthemes	Example Quotations
Health	Health Conditions Benefits of Health Healthy Behaviors	*Patient*: *“I have a [family]* *history of diabetes*, *my brother also has diabetes*.*”* *Patient*: *“It’s not funny when you’ve got really heavy kids who can’t walk you know*. *You’ve got diabetic kids*. *So it’s not as funny anymore*, *you know*.*”* *Patient*: *“After my mother died*, *I was scared to go back to sleep 'cause I said maybe I'll die in my sleep*. *I'm worried all the time*.*” Researcher*: *“About dying*?*” Patient*: *“Yeah”* *Patient*: *“Well you have to be active to get around and you have to be healthy to be active*.*”* *Patient*: *“The most important thing about health is nutrition*.*”* *Employee*: *“I don’t want to be in a wheel chair at the age of 40*, *because I was doing the wrong thing*, *smoking and drinking alcohol*, *eating junk food*.*”* *Employee*: *“health is important because you want to reach an older age”*
Healthy Environments/Union Community Health Center	Environments that encourage health	*Employee*: *“Once you leave* *[the health center]*, *there is nothing [healthy]*. *There’s* *pizza*. *There’s Popeye’s*. *There is crap all around*, *which makes the area sick*.*”* *Employee*: *“Drinking soda is the norm because that’s what you grew up with*.*”* *Patient*: *“The parents have to be the one to be the role models”*
Sugary Drinks—Behaviors and attitudes	Usage and patterns of sugary drinks Activities Emotional state Soda brand taste and preference	*Employee*: *“I know that soda* *is not good for me for many reasons… but once in a while I take Pepsi*.*”* *Employee*: *“And then I’ll pop another can*, *‘cause when it’s hot*, *it’s like really hot and you’ve got the air conditioner*, *it feels good with popcorn*, *you know*.*”* *Employee*: *“And I drink soda*, *like I share like a half a can like in the afternoon*, *me and*, *you know*, *my friend*, *or if my mother comes over*.*” Interviewer*: *“And your mother will come over and you’ll split just one soda*.*” Employee*: *“Yes*, *we just split it*.*”*
Sugary Drinks	Family and Friends	*Employee*: *“My mom is* *coming over this weekend and she loves soda and I don’t have soda in my house…*.*I think I’m going to have to buy it and I don’t want to*.*” Interviewer*: *“So you’re kind of conflicted*.*”* *Employee*: *“See I give in and I buy it…No*, *you buy it*. *Target has it on sale*. *You get three cases*, *you know*.*”* *Patient (of her son)*: *“I let him drink sodas*, *juices*. *And besides that the sugar—my grandfather recently died of diabetes*. *I don’t want him to get used to that either*. *And like I said*, *it’s not the best example*. *I am now trying to teach him because water is better*, *and that makes him feel better*.*”*
Sugary Drinks	Thoughts about SSB Free Zone	*Patient*: *“That would be a beautiful thing*. *But here's the thing*. *People got to educate themselves and a lot of people refuse to do that*.*”* *Employee*: *“There will be push back*.*”*
Health	Health Conditions Benefits of Health Healthy Behaviors	*Patient*: *“I have a [family]* *history of diabetes*, *my brother also has diabetes*.*”* *Patient*: *“It’s not funny when you’ve got really heavy kids who can’t walk you know*. *You’ve got diabetic kids*. *So it’s not as funny anymore*, *you know*.*”* *Patient*: *“After my mother died*, *I was scared to go back to sleep 'cause I said maybe I'll die in my sleep*. *I'm worried all the time*.*” Researcher*: *“About dying*?*” Patient*: *“Yeah”* *Patient*: *“Well you have to be active to get around and you have to be healthy to be active*.*”* *Patient*: *“The most important thing about health is nutrition*.*”* *Employee*: *“I don’t want to be in a wheel chair at the age of 40*, *because I was doing the wrong thing*, *smoking and drinking alcohol*, *eating junk food*.*”* *Employee*: *“health is important because you want to reach an older age”*
Healthy Environments/Union Community Health Center	Environments that encourage Health	*Employee*: *“Once you leave* *[the health center]*, *there is nothing [healthy]*. *There’s*
	Environments that discourage health	*pizza*. *There’s Popeye’s*. *There is crap all around*, *which makes the area sick*.*”* *Employee*: *“Drinking soda is the norm because that’s what you grew up with*.*”* *Patient*: *“The parents have to be the one to be the role models”*
Sugary Drinks—Behaviors and attitudes	Usage and patterns of sugary drinks Activities Emotional state Soda brand taste and preference	*Employee*: *“I know that soda* *is not good for me for many reasons… but once in a while I take Pepsi*.*”* *Employee*: *“And then I’ll pop another can*, *‘cause when it’s hot*, *it’s like really hot and you’ve got the air conditioner*, *it feels good with popcorn*, *you know*.*”* *Employee*: *“And I drink soda*, *like I share like a half a can like in the afternoon*, *me and*, *you know*, *my friend*, *or if my mother comes over*.*” Interviewer*: *“And your mother will come over and you’ll split just one soda*.*” Employee*: *“Yes*, *we just split it*.*”*
Sugary Drinks	Family and Friends	*Employee*: *“My mom is* *coming over this weekend and she loves soda and I don’t have soda in my house…*.*I think I’m going to have to buy it and I don’t want to*.*” Interviewer*: *“So you’re kind of conflicted*.*”* *Employee*: *“See I give in and I buy it…No*, *you buy it*. *Target has it on sale*. *You get three cases*, *you know*.*”* *Patient (of her son)*: *“I let him drink sodas*, *juices*. *And besides that the sugar—my grandfather recently died of diabetes*. *I don’t want him to get used to that either*. *And like I said*, *it’s not the best example*. *I am now trying to teach him because water is better*, *and that makes him feel better*.*”*
Sugary Drinks	Thoughts about SSB Free Zone	*Patient*: *“That would be a beautiful thing*. *But here's the thing*. *People got to educate themselves and a lot of people refuse to do that*.*”* *Employee*: *“There will be push back*.*”*
Health Literacy	Information Education	*Employee*: *“To have people* *to really get into the habit of cutting down on soda*, *we should have … signs of the effect that it causes on a person’s health when they use the soda* .* *.* *. *That will help people to adhere to it*.*”* *Patient*: *“I always read everything that I put in my [body]*. *I've learned to read every ingredient that's in there*. *But I think that they should impose a rule for a healthier drink*.*”*

### Education: A point of differentiation between SSBs and tobacco

Union pediatricians and staff emphasized that they wanted the implementation to include “teachable moments”—opportunities to educate parents on the dangers of SSBs. This idea was reinforced by our research. We found that, for our audience, education and information is a critical component for an SSB-free policy to achieve community acceptance and support. Despite the many similarities between SSB and tobacco control,[23,24] this is an important area of difference; while the harms of tobacco are well understood and widely accepted, it is not currently the case with SSBs[25,26].

Each of our focus groups included someone affected by diabetes, and the health effects of SSBs were taken very seriously. Yet, participants were highly interested in receiving more information about these health effects. Each of our focus groups featured moments where participants asked the moderator questions about the dangers of SSB use. Participants wanted to know if brown soda is less healthy than clear soda, if energy drinks are healthier than soda, and if diet soda is good or bad. One participant asked if ginger ale was a healthier option than other soda, and another person asked whether corn syrup or fructose is the same thing as sugar. It would be hard to imagine a focus group today with participants asking so many questions about the harms of smoking.

Soda consumption was a part of the lives of each of our participants. A few carried soda into the focus group. Some reported being current users, while others stated that they had completely quit, or at least tried to reduce their consumption. Because family and friends were a common context for consuming sugary drinks, some expressed difficulty in breaking the habit of drinking soda while maintaining social connections. For example, one employee said that splitting a can of soda was a typical part of regular visits with her mother. These examples remind us that, as explored through social practice theory, restricting SSBs from this environment may do little to remove these products from participants’ lives.

We felt it was clear from our research that handouts (such as those from the local health department) should be available to help answer health-related questions about the dangers of SSB consumption. As one participant stated, “We should have signs of the effect that soda and candies, cakes and sweet pastries causes on a person’s health, which would help people to adhere to it.”

An organization creating an SSB-free zone should recognize that such a process would be implemented within a complex environment of mixed and inconsistent social signals. As an example, when we ordered dinner for the focus groups, sweet and diet soda were automatically included with the catered meal orders. Our research team was required to pay extra to receive water instead of soda.

### Role modeling: Critical to acceptability

Our focus groups also emphasized the value that role models can play in creating a culture of acceptability of SSB-free zones. Patients emphasized that habits come from parents and the community and that changing bad habits requires changes in the house and in the community. Patients and employees, both spoke about the importance of positive role models and the cycle of good and bad habits being perpetuated as parents pass on these habits to their children and their children after that. As a staff participant said, “Drinking soda is the norm because that’s what you grew up with.” In one of the patient focus groups, someone emphasized that “the parents have to be the one to be the role models.”

Participants also recognized the value of the role modeling by the entire health center staff in supporting patients—especially the parents of small children—for whom the intervention is primarily intended. Our research supported previous research indicating that role modeling is an important component to creating an SSB-free culture.[27]

### Subtraction plus addition leads to acceptance

Consistent with previous research showing that behavior change is more difficult when a feasible alternative is not available, participants in both employee focus groups expressed a desire for, as one person described it, “a lounge or a healthy beverage area where coffee, tea and water was available.” Others expressed the view that providing easy access to water should be a component of disallowing SSBs. These beliefs support previous evidence [28,29] that social marketing interventions perceived as “taking away” may be ameliorated when additional health promoting products are introduced. In one group, when the researcher asked, “if there was coffee available here you might be likely to get coffee?” the participant responded, “instead of soda, yeah.”

Participants’ request for opportunities to incorporate more attractive healthy refreshment options should be taken seriously. Along with coffee, these options can include attractive water coolers (perhaps infused with fruit), water bottle filling stations, and free reusable water bottles.

### Health—In and of itself—Is valuable

At the outset, participants were asked to state why health was or was not important. Their responses covered the benefits of a healthy life: longevity, quality of life and “being around,” being able to see their children and grandchildren grow. The value of connecting older to younger generations was expressed across all the focus groups and among both younger and older participants.

## Discussion

### Public health implications

Focus groups participants were mostly neutral, supportive, or enthusiastically supportive of a SSB-free Zone at the health center. While public health advocates can be keen to dismiss education as an effective public health tool,[30] our study indicated that education is essential to alleviating concerns about removing beverage choices in institutional settings as participants asked us for more information to support their own personal reduction in SSB use. Information campaigns, combined with some form of healthy product distribution, are likely to improve levels of support for the SSB-free policy intervention. In a recent commentary, Kansangra et al. evaluated the impact of the New York City Department of Health’s educational and mass media campaigns to reduce SSB consumption. The campaigns were part of a series called “Pouring on the Pounds,” which we used at the end of our focus groups to demonstrate the health effects of added sugar in sugary drinks. In a survey of 1200 New Yorkers, 450 reported seeing the ads and drinking less sugary drinks.

The targeted marketing of beverage ads to racial and ethnic minorities and low- income communities, specifically low-income children, are important to consider when assessing obesity-promoting influences in these neighborhoods[31]. Children’s exposure to SSB ads has been found to be higher in areas with higher proportions of black children in the population, even though this is not the case for ads promoting diet sodas or non-SSBs.[31]. In the Bronx; African-American and Spanish-speaking populations are exposed to more processed food commercials and advertisements than white residents[32,33]. Over the next decade, the marketing of sugar-sweetened beverages is expected to increase dramatically worldwide. Coca-Cola has outlined plans to invest more than $17 billion in Africa between 2015 and 2020 and $4 billion in China between 2015 and 2017 [34]. PepsiCo’s declared a $5.5 billion investment in India between 2015 and 2020 [34]. Research efforts should continue to research the effectiveness of campaigns and messages that help prevent small children, especially in poor communities, from drinking these beverages.

## Limitations

The study findings may not be generalizable to other populations due to the unique nature of the sample, which is limited to the urban population of the Bronx. Additionally, medical center staff may have been more knowledgeable about health and more prone to demonstrate healthier behaviors than the lay population in the study. Social acceptability bias may have occurred in our study as participants may have tailored their responses to fit in with the group. The researchers used a series of probes, to obtain more information from each group in accordance with focus group methodology.

## Conclusions

The research team concluded that education must be a central component of any policy to restrict sugar-sweetened beverages on the premises of Union Community Health Center. SSB-free policies for health centers and workplaces has enormous potential to minimize soda use,[35] but unlike tobacco, people may not know about the various adverse effects of sweetened beverages. While it is clear that education should be an important component to SSB-free zone policy, it is still not clear what kinds of health education message frames are most effective to complement the policy (i.e. weight gain, tooth decay, disease, ethnic targeting, etc.). Nevertheless, this research may serve to remind health institutions to consider additional environmental enhancements, such as free coffee, tea and water dispensers, as was suggested by our participants. An evidence-based combination of education and policy change seems to be most likely to result in sustainable behavior change in this important public health domain.

## Supporting information

S1 File Palmedo & gordon—Focus group transcripts—UCHC 2015. (DOC)
