# Supplementary material for: How to be SSB-free: Assessing the attitudes and readiness for a sugar sweetened beverage-free healthcare center in the Bronx, NY
Source: PLoS One. 2019 May 15;14(5):e0215127. doi: 10.1371/journal.pone.0215127 (PMC6519811; doi:10.1371/journal.pone.0215127)
Supplement: S1 File — (DOC) [file pone.0215127.s001.doc]

# CITY UNIVERSITY OF NEW YORK

# SCHOOL OF PUBLIC HEALTH

**Union Health Center Focus Group #1**

**June 3^rd^, 2015**

Ubiqus/Nation-Wide Reporting & Convention Coverage

22 Cortlandt Street, Suite 802 - New York, NY 10007

Phone: 212-346-6666 ⬩ Fax: 888-412-3655

# Union Health Center Focus Group #1

# What do you think of when you think about health?

FEMALE VOICE: I don’t know. Exercise is the first thing that comes to mind. It’s exercise, followed of course by eating healthy. None of which I do, you know, really.

FEMALE VOICE: I think about popping a vitamin in the morning.

FEMALE VOICE: Vitamins, ‘cause sometimes I forget to eat something healthy, or something nutritious, like you know what, the vitamin will have it for the day and I’ll be okay, or a V8 Splash [LAUGHTER].

# 2. Do you think it’s important to be healthy?

FEMALE VOICE: It is, ‘cause we can live longer and see our grand kids and grand kids, um, we can do more in life instead of being in a wheel chair by the age of 40 and 50. We - - to move on like our ancestors, and live until 100 and something years old and talk about life and how beautiful it is.

INTERVIEWER 1: That was beautifully said. What else, why is it important? What’s important about health? Or is it not important?

FEMALE VOICE: No, see, I think health is important exactly for that reason, because you want to reach an older age. You want to get there, but you want to get there in a healthy way, where you’re going to, you know, sort of outlive, you want to outlive your parents and your grand parents. If they live to be 50 or 60, you want to live a little bit longer than that. You want your children to live longer than that, to sort of lead by example.

INTERVIEWER 1: Um-hmm, longevity, D.

FEMALE VOICE: Your grand kids, to see you grandkids.

INTERVIEWER 1: Grand kids, see your grand kids, yeah, anything else to add V?

FEMALE VOICE: I think it’s important to mind your health and what you eat and how you carry yourself on a daily basis.

INTERVIEWER 1: Why?

FEMALE VOICE: Because it’s your personal improvEnt of living well.

INTERVIEWER 1: Um-hmm, and just to push you even more, why is that important? Why is it important to live well, to be healthy?

FEMALE VOICE: Well to have a healthy lifestyle, and, um, like the girls said, to live longer, and then to see the younger kids survive and teach them how to be healthy too.

INTERVIEWER 1: Yeah, please, Crystal.

FEMALE VOICE: I wouldn’t mind being 60 and trying to run in a marathon.

INTERVIEWER 1: Oh really.

FEMALE VOICE: I wouldn’t mind doing that.

INTERVIEWER 1: Yeah, yeah.

FEMALE VOICE: But you have to be healthy for that.

INTERVIEWER 1: Yeah you can’t be unhealthy all the way up until 60 and then say now I’m going to be healthy and run a marathon.

FEMALE VOICE: I don’t want to be in a wheel chair at the age of 40, because I was doing the wrong thing, smoking and drinking alcohol, eating junk food with my sister, and everything is just breaking down inside me, and walking around with a diaper. I don’t want that.

[LAUGHTER]

INTERVIEWER 1: Okay, thank you. So when you think about, and again, coming back here at Union Community Health Center and you think about health here at Union coming out, you have physicians and services right, programs, community programs. What about the health center itself? What about the way the health center is and is laid out, and just the operations of the health center?

FEMALE VOICE: It’s - - .

INTERVIEWER 1: Yeah,

# 3. What is healthy, or not healthy about Union Community Health Center?

FEMALE VOICE: And we are very clean. We spray everything down. There’s so much viruses and bacteria. And there’s a lot of people that lie to us and tell us they don’t have anything. So without taking that chance we prefer to be more cleaner than anything, than dealing with later on, oh my God. You know.

INTERVIEWER 1: Yes, yeah, so cleanliness is something.

FEMALE VOICE: DefinitE..

INTERVIEWER 1: Yeah, hygiene.

FEMALE VOICE: And it’s sterile, everything is super clean.

INTERVIEWER 1: Everything is sterile, yeah.

FEMALE VOICE: Then as far as here, I mean I’ve been here for five months so far. I was here for when they switched over the vending machine out front, and switched everything to diet and water, which was a massive improvEnt over the sodas and stuff, because, you know, if you’re, at home we do not drink soda or juice. It’s just water, or you drink nothing. So, you know, it’s good to have it at work as well.

INTERVIEWER 1: Yeah, when that happened, when that vending machine switched over—

FEMALE VOICE: [Interposing] There was an uproar.

INTERVIEWER 1: Was it?

FEMALE VOICE: Yeah, there was an uproar. When people went to get soda, or I guess Snapple, or whatever, and all the Snapple is like Diet Snapple, yeah, people weren’t happy. But they’ve gotten used to it.

INTERVIEWER 1: Uh-huh, uh-huh, and then, was there an announcEnt or anything? Or was it just like one day?

FEMALE VOICE: Yeah, it was just one day, yeah.

INTERVIEWER 1: That’s something that is clearly an issue of the environment that, again, in public health we think about that. We, in public health we’re always talking about the environment that encourages or discourages health, making health the easy choice or the difficult choice. And in medical care often when they think about health they think about medical care, which is treating people, treating disease, which is obviously very important. But what you’re talking about is an example of again—

FEMALE VOICE: [Interposing] Yeah, and it’s surprising that it was so long, because every, any other hospital that you can go into, they don’t have any, everything is diet, low fat, or water in their vending machines.

INTERVIEWER 1: Is that right? Oh, okay.

FEMALE VOICE: Yeah, like if you go into Einstein or Montefiore you’ve never noticed it, but if you’ve ever gone to visit someone who is in the hospital, you have to go to the vending machine. Everything is diet or low fat. Same thing with schools, if you go into any school that has a vending machine everything is low fat or diet because it’s, you know, it’s the norm now I guess, you know.

# 3a. Anything about this health center, things that you think are very healthy, like the hygiene, or things that maybe could be better?

FEMALE VOICE: I think like surveying the public and you notice that they all at this point in time, whatever function you go to and you look around and you see people that were so thin at one time, they are all like double their sizes.

INTERVIEWER 1: So I’m sorry. You’re talking about like over the course of decades of whatever. Like you’re saying that—

FEMALE VOICE: [Interposing] No, let’s presume like five years ago.

INTERVIEWER 1: Okay.

FEMALE VOICE: I have known some and they were like really, really thin. And now looking at them, they have developed all of that weight, and you know. You’re looking to a trend to say oh my God, what are they doing that they developed all of that weight, you know?

INTERVIEWER 1: What do you think? Do you have any suggestions of ideas, theories as to why kind of happened?

FEMALE VOICE: I think that after a certain age people just don’t care how they eat and what they eat. And they just eat, you know, or drink, whatever. But the best thing I think would be for us to encourage them to be more sugar free, or a lot of water. Or even if they, because I’ve noticed also a lot of diabetics, they just want that sweet, and sweet, and sweet. And you know, like put it half and half, half soda and half water. You know, use seltzer water with - - water, you know.

INTERVIEWER 1: Um-hmm, so you also come back to the drinks. That’s an issue for you too.

FEMALE VOICE: Yes, yes.

INTERVIEWER 1: That seems like an important thing too, and, uh, we did talk about, and we mentioned that we were going to be talking about - - . We haven’t heard from you yet D, thoughts about health at Union Community Health Center, things that, you know, like Crystal said, there are things that they do, you know. And then - - and both Millie and V talked about these issues around the vending machine, the environment.

# 3b. Do you have any, you know, the environment being like the vending machine?

It was one way and then it was changed taking out some of the sugar. That’s a change in the environment.

# 3c. So anything else around here that encourages health, or not, or could it be better?

FEMALE VOICE: They should do a coffee lounge.

INTERVIEWER 1: They should do a what.

FEMALE VOICE: A coffee lounge {LAUGHTER].

INTERVIEWER 1: What do you mean? Why?

FEMALE VOICE: Because it’s, I don’t know. I’m more of a, I’m a coffee, or a tea drinker in the morning.

INTERVIEWER 1: Uh-huh.

FEMALE VOICE: They don’t have that here. Other hospitals have like a certain coffee lounge where you get like a bagel with cream cheese. I know that’s fattening but [LAUGHTER]—

INTERVIEWER 1: [Interposing] Okay, okay, keep going. Keep going.

FEMALE VOICE: Or little healthy muffins, or a green tea in the morning.

INTERVIEWER 1: Yes, really.

FEMALE VOICE: That would be awesome to have. Especially, it would be free for just us employees.

INTERVIEWER 1: Do you think they’d do that for free? I mean there are places where you can go in and get a free—

FEMALE VOICE: [Interposing] They should.

INTERVIEWER 1: --coffee or, or—

FEMALE VOICE: [Interposing] I know Google they do that.

[LAUGHTER]

INTERVIEWER 1: Google makes a lot of money.

FEMALE VOICE: Yeah.

INTERVIEWER 1: But, but, but you do know that, and you have heard that then.

FEMALE VOICE: Yes.

INTERVIEWER 1: You’ve heard about this idea of a coffee lounge. By the way, coffee, the research keeps coming out how coffee is actually really good for you.

FEMALE VOICE: Yeah it’s—

FEMALE VOICE: [Interposing] Well it depends what’s in it.

INTERVIEWER 1: As long as you don’t put anything else in it.

[LAUGHTER]

INTERVIEWER 1: - - sugar and cream - - .

FEMALE VOICE: You know, because in January it’s good for you, and then in June, oh no, it’s not good for you.

[LAUGHTER]

FEMALE VOICE: So you know, it depends what time of year it is.

FEMALE VOICE: [Interposing] Like more healthy, like a spa, we should have a spa here.

INTERVIEWER 1: [LAUGHTER] Well there you go. You have—

FEMALE VOICE: [Interposing] –a back massage—

INTERVIEWER 1: [Interposing] You’ve got great idea, but still—

FEMALE VOICE: [Interposing] –pink pedicures, manicures—

INTERVIEWER 1: [Interposing] You may need to work at Google actually.

[LAUGHTER]

INTERVIEWER 1: We’ll worry about that another time. So I, just - - the one person who had, and if you don’t have anything to say at this point that’s fine too, but is there anything?

FEMALE VOICE: No, nothing.

INTERVIEWER 1: Okay, no problem, so great, well that was very interesting to hear from all of that stuff. Um, now, um, what about, the next question I have to ask you is

# 4. what about soda and sugary drinks? What do you think about those?

You’ve said a few things about them, and I think you did too V.

# 4a.Let’s hear from all of you on your thoughts about soda drink. Do you drink them? Do you like them?

You’re smiling.

FEMALE VOICE: I was addicted to soda for a very long time until I got, I gained a lot of weight, and then I cut it off and I lost so much weight after my soda.

INTERVIEWER 1: When was that?

FEMALE VOICE: That was like probably a year ago.

INTERVIEWER 1: Uh-huh, and was it hard to give it up?

FEMALE VOICE: It, yeah, ‘cause you’re used to going home and opening a can of Ginger Ale, so I’m just drinking that cold ginger - - .

INTERVIEWER 1: You had a habit of, and it was at home. One of my questions is, that I would like to ask is how do you drink it and then how,

# 4b. what are your patterns if you do drink it (soda)?

So for you it used to be that you didn’t drink it necessarily here at work. Well did you bring it in with you to work?

FEMALE VOICE: I would buy cases at home, bring some here, some of the boxes.

[LAUGHTER]

INTERVIEWER 1: Right, right, and then you’d store it somewhere.

FEMALE VOICE: And then I’d store it in the fridge and just when you get thirsty running back and forth working you pop out a can.

INTERVIEWER 1: Right, pop a can, and when would you do that, at certain times a day, or was it like always around 2:00, or just when—

FEMALE VOICE: [Interposing] It could be sometimes at lunchtime, and then I’ll bring an extra can around probably 3:00 or 4:00. Right when we’re about to leave, you take that little quick [SIPPING NOISE].

INTERVIEWER 1: Uh-huh, and then eventually you get home.

FEMALE VOICE: And then pop another can.

INTERVIEWER 1: Pop another can, and then at some point you said—

FEMALE VOICE: [Interposing] And then I’ll pop another can, ‘cause when it’s hot, it’s like really hot and you’ve got the air conditioner, it feels good with pop corn, you know.

[LAUGHTER]

INTERVIEWER 1: By the way you’re describing it now it sounds like you still like it, but you don’t. You stopped it.

FEMALE VOICE: I don’t drink it. I don’t drink it.

INTERVIEWER 1: You stopped.

FEMALE VOICE: Yeah, I drink iced tea now. It’s sad. I don’t have the bubble feel.

[CROSSTALK]

[LAUGHTER]

INTERVIEWER 1: What about the rest of you?

# 4c. What are your opinions on soda - you’ve already expressed some – about soda, sugar drinks and, or your practices? What do you drink?

- - V - - .

FEMALE VOICE: At one time I used to drink a lot of coffee, but then it started to bother my stomach eventually, and I couldn’t find anything to really, you know, help that indigestion I used to have. So I just cut it out and now I am with tea, you know, like just tea.

INTERVIEWER 1: Okay,

# 4d. What about the sugar stuff though? What about soda and, I think you mentioned that you put soda, I think you might have even said that - - drinks - - sports drinks. But then you mentioned juice in that same category right?

FEMALE VOICE: Yeah, both of those are no, nos at our house.

INTERVIEWER 1: At your house.

FEMALE VOICE: Yeah, it’s just straight up water. You know, I can’t control what they’re, you know, out of my sight but—

INTERVIEWER 1: [Interposing] Okay, can I ask you though, you said your kids are going to be in Seattle on their own. So they are kind of older I guess.

FEMALE VOICE: No, well one is 15, and the other one is 18.

INTERVIEWER 1: Okay so 15, and they’re both going to Seattle to do this.

FEMALE VOICE: Yeah.

INTERVIEWER 1: Okay, so you, does this no, no policy of yours, is it like a real formal, like we are sorry everybody. We are having a—

FEMALE VOICE: [Interposing] Well yeah, I just stopped purchasing it ‘cause you know I do the main purchasing. I go to the market. I, you know, I do the food shopping on the weekends, and, uh, you know, it was, that’s the norm, because that’s what you grew up with, you know. And I find it’s economical. So it’s like, uh, one of the things that, and this is off the subject sort of, but one of the things that frustrates me about working here, and when I first started working here was the area, the neighborhood. It’s so geared towards, you know, inexpensive, sugary, fattening, you know, all the stuff that makes you sick, that makes you need a health center that you have to go to, you know, versus where I worked at before. I worked in Columbus Circle. Anything that you wanted that involved Kale you can get.

[LAUGHTER]

FEMALE VOICE: You know.

INTERVIEWER 1: That’s where my subway is when I, we have temporary offices at the - - and it’s at Columbus Circle—

FEMALE VOICE: [Interposing] Yeah, and the Whole Foods is right there.

INTERVIEWER 1: So I know what you’re talking about.

FEMALE VOICE: Yeah, you have the Whole Foods right there where you can get any thing. And you know, you’d go in in the morning, get your salad, get your chia seeds, whatever it is that you’re eating that day. I’d make my own peanut butter, you know, the whole thing. So it was a total, you know, you’re eating healthy because that’s what was available to you. Where I would never have touched kale before, but that’s what’s there, so that’s what you eat. And you know then to sort of come here and it’s like so I bring my own lunch every day because otherwise, you know, I’d weight 300lbs at the end of uh—

INTERVIEWER 1: [Interposing] Once you go out the door and look for a place to eat—

FEMALE VOICE: [Interposing] You can’t. There is nothing. There’s pizza. There’s Popeye’s. There is crap all around, which makes the area sick, which makes, you know, you need this place. So I guess you know it sort of provides a job for us in a crazy circle.

INTERVIEWER 1: [LAUGHTER] But a sad story

FEMALE VOICE: A sad, you know, but it sort of goes hand in hand, I think, with poverty as well, where you know, a lower economic base, that you’ve got stuff that you can buy for three bucks. You can get a soda and maybe some chicken wings, you know. And it’s going to kill you, you know. Whereas, for three bucks, maybe if there was somewhere to get a salad, you know, you can, I mean, I don’t know. I don’t know.

INTERVIEWER 1: No that’s very good, very intriguing, that, what you just said is exactly what we do in focus groups. I mean really, you don’t get that data when you ask questions. So, uh, I want to ask you one more thing. You said something about you changed your, uh, you used to buy sodas.

FEMALE VOICE: Oh yeah, absolutE., all the time, because when we grew up that’s what we used to drink.

INTERVIEWER 1: Yeah, you said that before you would—

FEMALE VOICE: [Interposing] My mother used to buy soda all the time because, you know, it was like a treat for you in the beginning, and then you know, you sort of got into a different economic bracket where you could afford it all the time, so she used to buy it all the time, you know. And we were, I guess addicted to it, because that’s all you drank. Then you become an adult and you carry those habits over. You have your own kids. You carry those same habits. And so you read and you start figuring out, you know what, this isn’t so great for them. It’s not great for us. We need to change it. And so we did. And we did a solid turn around. And it was like you know, you’ve got soda, let’s finish it up, because come Monday, there is no more.

INTERVIEWER 1: Really, that, was it that cut and dry?

FEMALE VOICE: That cut and dry.

INTERVIEWER 1: And you rEmber it clearly, that situation.

FEMALE VOICE: [LAUGHTER] Yes.

INTERVIEWER 1: Now what about your kids? How did they react?

FEMALE VOICE: Oh they stomped, and they were not happy.

INTERVIEWER 1: Did they? Really, just like you said there was an - - .

FEMALE VOICE: Yes, but you know what, after a while you get used to it, and after awhile they reach for the Poland Springs. We got a water cooler, you know. We buy bottles of water. You keep them in the fridge, and they, that’s what they have to reach for, you know.

INTERVIEWER 1: Interesting, uh, when was that, how long ago?

FEMALE VOICE: Uh it’s going to be about two years.

INTERVIEWER 1: Really, wow, interesting. So I want to go back to you V, because you mentioned coffee. But

# 4e. I specifically want to ask you about sugary drinks, soda, sugary drinks. Do you drink them at all, or do you, does your family, community, anybody that you know?

FEMALE VOICE: [Interposing] Actually, where I came from, my mom, it was 15 of us, and my mom, she would make like strawberry flavored, like fresh strawberries, she would make strawberry drink. She would make the guava drink. She would make lemonade. She would make everything from scratch. And my mom would tell us, you know, that it’s not good to consume a lot of soda, because you could get nosebleed. She said you could get nosebleed, especially if the sun is always hot. So that is embedded in us from that time onwards. So coming, moving around, we usually buy like the liter soda. But I inherited in my children that, you know, it’s better to mix up half and half, even though my eldest daughter does not want to do that. And she still sometimes wants to drink the whole can of soda.

INTERVIEWER 1: Right, half and half being—

FEMALE VOICE: [Interposing] Half water and half soda.

INTERVIEWER 1: Half water and half soda, oh.

FEMALE VOICE: Um-hmm.

INTERVIEWER 1: And you do that.

FEMALE VOICE: Sometimes, not all the time.

INTERVIEWER 1: So sometimes you’ll do that, but this fresh soda, juice concoction that your mother did influenced you for the rest of your life.

FEMALE VOICE: Yes, the juices, yes, yes, I’d usually do that, yes.

INTERVIEWER 1: You do that too.

FEMALE VOICE: I give it to D sometimes too.

INTERVIEWER 1: Oh did you?

FEMALE VOICE: What?

FEMALE VOICE: The fruit juices that I make.

FEMALE VOICE: Oh yeah.

INTERVIEWER 1: So D, we’ve heard a lot of perspectives, you know, uh, people who drank it and then stopped, and the family stopped growing up. Where do you fit in with all that if I can ask?

# 4e. Do you drink soda, your history, your thoughts, your opinions?

FEMALE VOICE: I used to drink soda. I love ice cream. That helps my heartburn. So I still have ice cream, which you’re not supposed to. But I try to buy like the Skinny Cow or Weight Watchers. I don’t know if it’s going to help or what. It does help my heartburn, but I don’t know if it’s healthy for me. And I drink soda, like I share like a half a can like in the afternoon, me and, you know, my friend, or if my mother comes—

INTERVIEWER 1: [Interposing] Here for a break.

FEMALE VOICE: Yeah, for lunch.

INTERVIEWER 1: For lunch, okay, and half a, you each, you have one can. You split the one can.

FEMALE VOICE: Yeah, we split it. Yeah, the rest of the day we have water, you know. But you see, my son he works for Mixed Martial Arts. So he is on a strict diet, because he is fighting in August, which I wish he wouldn’t but—

INTERVIEWER 1: [Interposing] Really, but he must be incredibly fit and muscular right.

FEMALE VOICE: Yeah, but, you know.

[LAUGHTER]

FEMALE VOICE: It’s awful to see. I saw one fight already, and he eats very healthy. I mean, the only thing he doesn’t drink water. He has salads and—

INTERVIEWER 1: [Interposing] I’m sorry. You said he doesn’t drink water, or—

FEMALE VOICE: [Interposing] He drinks—

INTERVIEWER 1: [Interposing] He drinks a lot of water.

FEMALE VOICE: --plenty.

INTERVIEWER 1: Plenty of water, okay.

FEMALE VOICE: Plenty of water.

INTERVIEWER 1: Yeah, okay.

FEMALE VOICE: I mean gallons of water, he eats healthy, very well. So I’m starting also to eat healthy, because I’m trying to lose weight.

INTERVIEWER 1: But in relation to him it sounds like you’re kind of like patterning or like there is a relationship between how he eats and you.

FEMALE VOICE: Right, right, yes, yeah, well my husband, he was, I mean he was the healthiest person. He used to go to the gym. He used to be there three hours running, you know, doing this - - , you know, three hours at the gym. And he used to eat healthy, you know. He is no longer here, but, you know, and then my son also—

INTERVIEWER 1: [Interposing] He was influenced. It sounds like your son was influenced by his father.

FEMALE VOICE: Yes, yes.

INTERVIEWER 1: I’m sorry. I interrupted.

FEMALE VOICE: No, it’s okay. And so now I am starting to eat, you know, trying to watch what I eat.

INTERVIEWER 1: Right, so you’re actually influenced by your son—

FEMALE VOICE: [Interposing] It’s just, you know, one thing that bothers me—

INTERVIEWER 1: [Interposing] Please.

FEMALE VOICE: --you know, I mean, my husband was like the healthiest person, you know. And he passed away, and he was the healthiest person. He was going to the gym. He ate well. And I just don’t understand that.

INTERVIEWER 1: Can I ask how he passed?

FEMALE VOICE: He had a betel cell carcinoma. That’s how it started.

INTERVIEWER 1: How long ago was that?

FEMALE VOICE: Two years, two years ago.

INTERVIEWER 1: And you have a close relationship with your son it sounds like.

FEMALE VOICE: Yes, well he was very close to—

INTERVIEWER 1: [Interposing] His father.

FEMALE VOICE: Yes he was very close to his father. His father used to go to the gym. Now he, I guess he watched what his father used to do.

INTERVIEWER 1: Sure.

FEMALE VOICE: His father used to go to the gym. He goes to the gym. His father eats healthy. He eats healthy. And now I’m starting also to try to, you know, eat healthy.

INTERVIEWER 1: And again, I’ve said this a couple of times, but you know, it sounds to me like, you say you started, but it sounds like your son, you and your son, you know, you were influenced by his healthy—

FEMALE VOICE: [Interposing] By him, yes, exactly.

INTERVIEWER 1: Okay, so if I can bring it back to the soda and the sweetened drinks, what’s that, how has that

# 4f. what has that pattern been like for you? Is that something that you used to drink (soda) and you don’t drink as much?

You split the one drink—

FEMALE VOICE: [Interposing] I used to drink soda, you know, like I love sweetened iced tea. But now I—

INTERVIEWER 1: [Interposing] But you do have the one half a drink with, as a ritual kind of.

FEMALE VOICE: Yeah, that half a drink.

INTERVIEWER 1: You have a friend, a coworker that you split it with.

FEMALE VOICE: Yeah, yeah, and when my mother comes over we’d have half.

INTERVIEWER 1: Oh, oh, I’m sorry. I thought you were saying you—

FEMALE VOICE: [Interposing] Even here.

INTERVIEWER 1: Even, oh, she comes over here.

FEMALE VOICE: She comes where I am, yeah.

INTERVIEWER 1: Oh, she’ll come. Your mother would come over here.

FEMALE VOICE: No, no, at my house.

INTERVIEWER 1: At your house, okay, I’m sorry.

FEMALE VOICE: Yeah, we’ll have half.

INTERVIEWER 1: Uh-huh.

FEMALE VOICE: I’ll have half a soda.

INTERVIEWER 1: You’ll have half a soda. And so on weekends, or whatever, when your mother comes over—

FEMALE VOICE: [Interposing] A day, a day, a day.

INTERVIEWER 1: A day.

FEMALE VOICE: Yeah.

INTERVIEWER 1: She’ll come over and you’ll split just one soda.

FEMALE VOICE: Yes, we just split it.

INTERVIEWER 1: And why only a split, why would you not each have one, because you—

FEMALE VOICE: [Interposing] Uh, it’s too much, too fattening, too much sugar.

INTERVIEWER 1: Yeah, Crystal, you’re—

FEMALE VOICE: [Interposing] You can become a diabetic.

INTERVIEWER 1: Yeah, okay, Crystal, you’re nodding your head, I mean, because you—

FEMALE VOICE: [Interposing] It’s true. I drank so much soda. I lost so much weight when I stopped drinking soda. And I didn’t believe that they said it was the soda. And so I stopped. And when I stopped I went whoa. I’m in skinny jeans again.

[LAUGHTER]

INTERVIEWER 1: Now, um, again, one of the things that’s interesting about soda, and as people who are concerned about this, or want to research this idea of how people drink soda, why and how, is exactly this question of when and how. So that’s why I’m asking you these questions about it. When your mother comes over it’s something you look forward to, but it’s like this little, all right, you pack your Coke. You take it out - - and there is something nice about that right. It’s like you’re breaking bread or whatever right.

FEMALE VOICE: She likes the ginger ale so—

INTERVIEWER 1: [Interposing] She likes the ginger ale. And that’s—

FEMALE VOICE: [Interposing] See, it’s a good thing that we split it.

FEMALE VOICE: No, I got to drink the whole can.

[LAUGHTER]

INTERVIEWER 1: And you got to drink a can or even more.

# 4g. So before you gave it up did you feel like it was something that was just habitual that you just did, or did you actually like it?

FEMALE VOICE: Oh God I loved it.

INTERVIEWER 1: Tell me about that.

FEMALE VOICE: But you know what’s sick.

INTERVIEWER 1: Please.

FEMALE VOICE: I love diet soda. That’s all I drank.

INTERVIEWER 1: And you stopped doing that too.

FEMALE VOICE: I stopped doing it.

FEMALE VOICE: They say that’s no good. Is that true?

FEMALE VOICE: That’s a lot of why I stopped doing it, because all, like I said, you start reading and you learn, and it’s like, you know, all the chemicals and everything, it’s really not good for you and you know.

INTERVIEWER 1: Yeah, Mille is right. I mean there is research that shows that there are some adverse relations. Even with obese, I mean even in actually weight gain and diet, because there are these other chemicals in there that act differently than sugar does, but also can be what they call obese - - .

FEMALE VOICE: Even the foods, there is stuff in it.

INTERVIEWER 1: Processed foods.

FEMALE VOICE: The frozen foods.

INTERVIEWER 1: Frozen foods are processed, and that means they take out a lot of the fiber, and that makes it - - .

FEMALE VOICE: They have to put other stuff in it so - - .

[CROSSTALK]

FEMALE VOICE: I loved my diet soda. It was that and cigarettes you know.

FEMALE VOICE: I stopped that too.

FEMALE VOICE: Yeah.

[LAUGHTER]

FEMALE VOICE: The other thing is, my mom is coming over this weekend and she loves soda and I don’t have soda in my house.

INTERVIEWER 1: What do you do when she comes over? Do you tell her no, or do you say I’m going to get some soda just for you?

FEMALE VOICE: No, I just tell her listen, there’s iced tea right there’s [LAUGHTER]—

INTERVIEWER 1: [Interposing] So you don’t say well I don’t drink soda, but I’m going to go out and buy some soda for my mom.

FEMALE VOICE: She asked me to.

INTERVIEWER 1: She asked you.

FEMALE VOICE: I think I’m going to have to buy it and I don’t want to.

INTERVIEWER 1: So you think you’re going to have to buy it and you don’t want to. So you’re kind of conflicted.

FEMALE VOICE: See I give in and I buy it for when we have, like two weekends ago we had a barbecue for Memorial Day. What am I going to tell everyone who shows up? Sorry, no soda because we don’t drink it. No, you buy it. Target has it on sale. You get three cases, you know.

FEMALE VOICE: Me too, I get it in Target.

[LAUGHTER]

FEMALE VOICE: Three for $11, so you know.

[LAUGHTER]

INTERVIEWER 1: Even though you don’t but it you know the price. And it sounds like you are—

FEMALE VOICE: [Interposing] I have to buy it this weekend. I have no choice.

INTERVIEWER 1: --you’re in the same thing. And you’re going to. But you, okay, you, well you say no choice the first, the second you were thinking well maybe I should stand up—

FEMALE VOICE: [Interposing] My mom.

INTERVIEWER 1: It’s your mom. It’s not going to kill you.

INTERVIEWER 1: I don’t see her, like once, I can see her once in a blue.

INTERVIEWER 1: So if you didn’t buy it, and your mom came over, it would be an issue. She would be like where is the soda right.

FEMALE VOICE: Well if I see my mom once in a blue, I would accommodate her. I would buy her the soda. But if it’s like, I see her every weekend, no.

[LAUGHTER]

INTERVIEWER 1: Because it’s a special thing, just like Memorial Day weekend. It happens only ever so often.

FEMALE VOICE: Yeah.

INTERVIEWER 1: So were any of you going to say anything else about this? V you didn’t say too much, but you did say some interesting things about the history of your growing up, the influences that influenced your, uh, opinion and practice. So that’s great to hear this. This is very interesting. Now as I said, from a health perspective, there is some concern, increasing concern about this. And Union is concerned, and that’s why they got rid of the vending machine. Obviously - - . Other people noticed it too. And there is a lot of sugar in these drinks, and medical doctors are very interested in getting people to drink less.

So what do you think about that? What do you think about, let’s go ahead and just go all the way to say the idea of Union Community Health Centers got rid of the machine.

# 5. What if they said something like we would like to have no one drink soda in this building?

You know, there are adverse health effects, and I’ll show you actually some videos, but there actually is some research that indicates there is a connection between the consumption of soda and not just, you mentioned weight gain, but, um, we know diabetes, right, that’s not surprising, but heart disease, and cancer, and even potentially brain function.

So these are things that the medical community, the physicians here at Union Community Health Center have brought this up, and that’s why - - . And they are talking about the idea of, it’s like we don’t allow people smoke in this building. We don’t allow people to drink alcohol. What if they took, applied those rules to soda? What would you guys think?

FEMALE VOICE: I have a question. So they’re trying to make soda like cigarettes, like you’re not supposed to smoke anymore, kind of thing.

INTERVIEWER 1: That’s a question you’re asking me.

FEMALE VOICE: Yeah.

INTERVIEWER 1: There’s a movEnt within the medical community that says not so much, you know, we should make soda illegal or whatever, but for example, there are things like, public health people, professionals, scholars and researchers who work at universities, like myself, are talking about suggesting that there maybe should be things like warning labels. So drinking too much, that would be one example. Restricting advertising for kids, no advertising at all on certain TV shows. Just like you can’t put a, you can’t have a secret commercial. If the cigarette companies really want to have their way they would have cigarette ads on those TV shows that 14 year olds, ‘cause that’s the perfect age to get them, and some people are saying well maybe we should apply those same standards to soda. This is very new. You said something like well, you know, it’s the wave that’s going on now. Einstein you said, they don’t have it. Um, but in some ways it’s a very new wave too.

FEMALE VOICE: Oh it’s absolutE. new.

INTERVIEWER 1: Some people are talking about warning labels, like restricting advertising. That’s a new thing. And so that’s what, I also want to ask you about that question.

# 5a. What do you think about what I just said? Those ideas of being more restricting, having more rules, you have rules in your house, what about rules at a place like a community center?

FEMALE VOICE: If you look at it as a big picture thing then you’re okay with it. If you look at it though, there’s got to be some push back, because who are you to tell me I can’t drink soda? What if I want to drink soda? It’s not killing me, and it’s not illegal. So why can’t I have it? It’s bad for my health. That’s a decision that I’m willing to make, the risk I’m taking. So there will be push back.

INTERVIEWER 1: You just described the two sides of the argument. If I may ask, where do you fall into that?

FEMALE VOICE: I don’t know.

INTERVIEWER 1: You don’t know. V you look like you were about to say something. You were kind of shaking your head or something.

FEMALE VOICE: I think in an environment to have the people to really get into the habit of cutting down on soda, we should have like really signs of the effect that it causes on a person’s health when they use the soda or too much sweet, not only elaborating on soda, but most of sweet sugary stuff. That will help people to like really, you know, adhere to it.

INTERVIEWER 1: Put posters or something that says sugar will cause these, but not necessarily say don’t drink soda.

FEMALE VOICE: No, not really sugar will cause it, but any sugary stuff, or so, which is like soda.

INTERVIEWER 1: Including sodas.

FEMALE VOICE: Yeah, you know, like candies and cakes and sweet pastries and stuff like that.

INTERVIEWER 1: It sounds like what you are saying is getting information out to people is important.

FEMALE VOICE: Yes.

INTERVIEWER 1: But not necessarily telling you that you can’t do it.

FEMALE VOICE: No, exactly.

INTERVIEWER 1: But just give the information. Where do you fit in Crystal, D on this?

FEMALE VOICE: Isn’t too much of something bad for you anyway?

INTERVIEWER 1: Right.

FEMALE VOICE: Too much of anything is bad for you.

INTERVIEWER 1: Yes.

FEMALE VOICE: Wouldn’t people like see it like sort of that too, the same way as everything else?

INTERVIEWER 1: Well Millie really did say it very eloquently when she said from a big picture perspective, we’re seeing in public health, well we, in public health you study the big picture. You study the full population data. So you look at rates of, you know, diabetes is going up. Childhood obesity is going up. That’s what public health looks at. From an individual perspective, upstairs in the pediatric unit they are seeing more kids, a pattern of more childhood diabetes coming in. So that’s the patterns.

So you said well isn’t it your individual choice? But they’re saying what do we do about these patterns? V said maybe we should have more information right.

FEMALE VOICE: Yes.

INTERVIEWER 1: What do you, I mean, so—

FEMALE VOICE: [Interposing] Why would you give soda to a child in the first place? I never give soda to kids.

FEMALE VOICE: People just do. It’s just something that you do anyway. I’ve seen soda in bottles, in baby bottles.

FEMALE VOICE: Yeah, I’ve seen that too.

FEMALE VOICE: Yeah, you see it all the time. So it’s like, you know, it’s something that people don’t even think about. Soda is a - - . You buy it. You give it to the kid. It keeps the kid calm. It’s not even something that anybody thinks about.

FEMALE VOICE: Maybe they need more education about soda.

FEMALE VOICE: Education absolutE., but it has to be something that is ever changing. We have such a short attention span that anything that you tell people, you need to tell them the same thing in a different way two weeks later. And two weeks after that it has to be the same message in a different, ‘cause no, it’s an X-box, Microsoft world where nobody pays attention to anything for more than 20 seconds.

FEMALE VOICE: That is true.

FEMALE VOICE: You lose everyone’s attention. Like those commercials that they had when the mayor was trying to ban soda sales, you know, over 20ozs let’s say, you know, that was such a big deal, and people were pissed, and they were buying, you know, two liter bottles, just like I’ll show you.

INTERVIEWER 1: [LAUGHTER] - - was - - .

FEMALE VOICE: You know, exactly, like you jerk, you can’t take my soda away, you know. And after awhile that sort of died down because the message was sort of coming through. It’s not funny when you’ve got really heavy kids who can’t walk you know. You’ve got diabetic kids. So it’s not as funny anymore, you know. So people started understanding, you know, maybe soda is really bad for you. So a little bit of that is getting through. But you know that message dies away.

INTERVIEWER 1: The message dies away so that, if I can push back, push on you a little bit. Education may be, it not be enough. Maybe if you give all these people this information—

FEMALE VOICE: [Interposing] I think it should be a constant reminder, especially when the parent takes the kids to their appointments, you know, let the doctor try to like, advise them, because you cannot make them understand if they don’t want to understand. But let the doctors constantly advise the parent, you know. If you love your child just make sure, you know, you do that, or do that. But then the pattern comes from home too. If the children see the parents are drinking soda, mommy can I have some? No you can’t. Why? Because you can’t, I said no you can’t. Then show example, you know, instead of drinking soda just drink water you know. Look mommy is drinking water. You should be drinking water, because it’s going to make you healthier.

INTERVIEWER 1: But that’s not happening right now.

FEMALE VOICE: No, like I said, it has to be a constant reminder. And the parents are the ones that have to educate the siblings, you know, the children, or whoever is in the household

INTERVIEWER 1: You know, you asked Crystal why would you give a kid soda, but people are giving their kids soda. And so what—

FEMALE VOICE: [Interposing] Yeah, it’s cheap and plentiful.

INTERVIEWER 1: It’s cheap and plentiful, exactly.

FEMALE VOICE: - - .

INTERVIEWER 1:

# 5b. So is making it less cheap and less plentiful a solution? That’s the question that we’re dealing with in public health right. Is education enough? It is what do we do? How do we deal with this issue? Is just education, is the doctor educating the parent at that time of the obesity, at the time of the diabetes enough?

FEMALE VOICE: They’re not listening.

INTERVIEWER 1: They’re not listening.

FEMALE VOICE: They’re not listening.

INTERVIEWER 1: So what do we do?

FEMALE VOICE: You can’t make soda a luxury item. That will never happen. Pepsi will never allow that.

INTERVIEWER 1: Um-hmm, right.

FEMALE VOICE: You know, soda is never going to be $5.00 for a 20oz. It’s never going to happen.

INTERVIEWER 1:

# 5c. So what if Union Community Health Center said this isn’t happening? We’re not - - their kids - - . We got to do something about it. Let’s just have no soda here.

FEMALE VOICE: Not allow it on the premises.

INTERVIEWER 1: Yeah.

FEMALE VOICE: When you come in you cannot have soda on you.

INTERVIEWER 1: Yeah, something like that, yeah.

FEMALE VOICE: I’m sure people will bitch about it.

FEMALE VOICE: Yeah. I don’t think—

FEMALE VOICE: [Interposing] But after awhile you get used to it.

FEMALE VOICE: But then they’ll hide it in their bags.

FEMALE VOICE: Oh yeah, they’ll hide it in their bags or whatever, but after a while, I think you get used to it. It’s like everything else where, you know, you can’t smoke in public places. We didn’t like that, but we got used to it.

INTERVIEWER 1: Right, the non-smokers, they liked it, but—

FEMALE VOICE: [Interposing] Yeah, but you know, you got used to it.

INTERVIEWER 1: Right, but you got used to it. And we’re now, it’s the norm - - .

FEMALE VOICE: Yeah.

INTERVIEWER 1: That’s not the issue. The issue is not the employees here. The issue is the patients, and how do we deal with that you know, and the fact that - - is like, educating. We talked about why would someone give their kid a soda and - - educate - - ? They are educated. They come to me and say we’re doing the best we can. We need to figure out how to step it up, okay. So here is four, yes, go for it.

FEMALE VOICE: How do the flyers - - ?

INTERVIEWER 1: Sorry.

FEMALE VOICE: Like flyers.

INTERVIEWER 1: Sure, sure, sure, yeah, of course, of course.

FEMALE VOICE: No, no, I’m saying they can like make—

INTERVIEWER 1: [Interposing] Right.

FEMALE VOICE: How about if they eliminate them from McDonalds or Burger King?

INTERVIEWER 1: If they, well, good luck telling McDonalds that.

FEMALE VOICE: That will never happen.

FEMALE VOICE: Yeah, that’s the hottest thing they sell.

INTERVIEWER 1: But what you’re talking about, that would never happen.

FEMALE VOICE: Yeah, exactly, but that’s what they buy every single day is McDonalds and Burger King with soda.

INTERVIEWER 1: Right, so what you’re talking about is what we’ve talked about in public health. It is somehow changing the environment. And that’s what that soda vending machine is doing. It changes the environment. There is education, and that’s what you are talking about—

FEMALE VOICE: [Interposing] You might have flyers like in the health facilities.

INTERVIEWER 1: Right posters that say, so—

FEMALE VOICE: [Interposing] Let them put like flyers, you know, like—

INTERVIEWER 1: [Interposing] Just the flyers.

FEMALE VOICE: Yeah.

INTERVIEWER 1: That says something about - - .

FEMALE VOICE: Yeah.

[CROSSTALK]

FEMALE VOICE: What about videos?

INTERVIEWER 1: I’m sorry.

FEMALE VOICE: How about videos? People like watching videos, especially on Facebook.

INTERVIEWER 1: Well let’s stick with, well, the problem is here in this building there is not a lot of opportunity to watch much videos. But let’s got ahead and, I’m going to take what you said, which is letting—

FEMALE VOICE: [Interposing] Yeah, they could, you know, just leave it on the counter, and people, we could just tell them to take a flyer.

INTERVIEWER 1: Okay, perfect set up. That’s a perfect setup for where we’re going.

FEMALE VOICE: That’s what I was thinking.

INTERVIEWER 1: [LAUGHTER] So now what do you see in your flyer?

FEMALE VOICE: You need to have pictures.

INTERVIEWER 1: Here are the four, huh.

FEMALE VOICE: You need to have pictures on them.

INTERVIEWER 1: You need to have pictures. Now without getting into the details of pictures and where you put them, that can happen later, and by the way, there is a health committee here at Union that’s going to probably deal with some of that stuff. And these are difficult. You really did put your finger on the fact that these are, all of you did. You talked about how easy is it to just rip out the, so these are very complex. And you also comment about human behavior, and human relationships, and, you know, and the systems that are, the environment around you when you walk out the door. All of these things are just so difficult right. They are really, really hard. If they weren’t hard, then they would have been better.

So getting to your point about flyers, and even video, getting back to what I was saying, let’s talk about these different approaches. What do you say on a video? And what do you say in a flyer right? How do you do it? So I’m going to talk to you, walk you through four kind of angles all right. And I want you to vote at the end on which one is the best. So here is the first one.

It is brain function, education and success. Soda and energy drinks contain more sugar than people should consume in a day and there is some recent scientific research to indicate that sugar damages brain function. Soda and sugary drinks may be affecting our children’s ability to do well in school, and their future education and success in life.

So what about a flyer that talked about that? And here is a video that kind of gives you some more examples. So L. is getting us read. You want to turn around, or you want to look at that one. Here she goes, all right, ready. Here we go.

# CITY UNIVERSITY OF NEW YORK SCHOOL OF PUBLIC HEALTH

**Union Health Center Focus Group #2**

**June 4, 2015**

**12:00 p.m.-2:00 p.m.**

Ubiqus/Nation-Wide Reporting & Convention Coverage

22 Cortlandt Street, Suite 802 - New York, NY 10007

Phone: 212-346-6666 ⬩ Fax: 888-412-3655

# Union Health Center Focus Group #2

MS. C: I think health is important to be active because now my grandmother is sick so she's not--and she doesn't want to do nothing. She wants to just sit in the house. She has - - . I think she has cancer of something. I don't know. And she doesn't want to do nothing. Vice my mom has breast cancer and she's like--she doesn't want to believe it, so she still thinks that she's all right and she's like--she wants to live normal, but my grandmother she feels like she's not healthy. She doesn't--to me it's like a mindset, a mind thing.

MR. INTERVIEWER: Yeah. So your mother who has breast cancer has a positive mindset you're saying.

MS. D: Like say for instance, the rehab department which is on the other side of the building they have a - - place right there as soon as you come in. They have the family practice in the back. In the afternoon they call it urgent care.

MS. C: I used to drink soda. Like I used to drink Pepsi but Pepsi to me started to get addicting. I don't want to be dehydrated. I drink a Pepsi and I just want it more and more, so I stopped. And then when I got pregnant they told me it's not good--

MR. INTERVIEWER: [Interposing] They did.

MS. C: --to drink soda, so.

MR. INTERVIEWER: Really?

MS. C: So I started drinking vitamin water and they told me that's not good either--

MR. INTERVIEWER: [Interposing] Okay.

MS. M: [Interposing] Why?

MS. C: --to drink vitamin water?

MS. M: I'm sorry.

MS. C: They--I don't know. My doctor told me it's when--it's like you call overdose on the vitamins 'cause I take the prenatal pills.

MS. M: Okay.

MR. INTERVIEWER: Okay. That's interesting.

MS. C: So now I drink juice. I drink Snapple and I need to start drinking water but--

MR. INTERVIEWER: [Interposing] Okay.

MS. D: That's the best.

MR. INTERVIEWER: Very interesting information there. S, are you still thinking?

MR. S: Well I'm diabetic also.

MR. INTERVIEWER: Okay.

MR. S: And I cut out the sodas and stuff and some labels on some beverage can be misleading 'cause some of them that don't directly say sugar.

MR. INTERVIEWER: Yes.

MR. S: It would say fructose--

MS. D: [Interposing] Yes.

MR. S: --lactose, glucose, and a lot of people like me doesn't understand what those words mean. So they said oh there is no sugar.

MR. INTERVIEWER: Okay.

MR. S: But they're misinterpret what these words mean.

MR. INTERVIEWER: Yeah.

MR. S: And they take and they give it to their kids and everything and even if they're diabetic 'cause they're not really understand basically what these words mean.

MR. INTERVIEWER: Okay. Yeah.

MR. S: So they look for the words sugar.

MR. INTERVIEWER: Right. You're saying--

MR. S: [Interposing] And a label should be--you know, they should put more, you know, details--

MR. INTERVIEWER: [Interposing] Right.

MR. S: --on the--

MR. INTERVIEWER: [Interposing] It would be easier for the average person to understand.

MR. S: Yeah, because everybody is not on the level academically.

MR. INTERVIEWER: Right, right. That's an issue what we call in public health literacy and it's true. You're right. Things are complicated - - .

MS. G: I have a question. I just want to know about green tea.

MR. INTERVIEWER: Yes.

MS. G: Is that healthy?

MR. INTERVIEWER: Actually it is very healthy.

MS. G: I never tried it or anything. I'm just confused.

MR. INTERVIEWER: Green tea is very healthy. There's a lot of research that shows when people have cancer a lot of times doctors tell them to drink green tea every day.

MR. S: It's also good for diabetic and to lose weight.

MR. INTERVIEWER: Yes. It's good for everything. But the problem--when you talk about green tea and coffee and those--and coffee has a lot of research that shows coffee is very healthy. But a lot of people think--you're looking at me like really.

MS. D: No, no. I'm not looking at you like--really I'm just waiting to see the next thing you're going to say.

MR. INTERVIEWER: The next thing I'm going to say is this. People say oh coffee is healthy, which it is, so then they have a coffee with three sugars and a bunch of half and half in there and they're drinking it two times a day. And they think I was told coffee is healthy, but it's all the stuff you put in with the coffee--

[Crosstalk]

MR. INTERVIEWER: Same thing if I tell you green tea is healthy but you have a bunch of sugar and a bunch of cream and then you drink a little green tea in there, right - - . Let's go back to the sugar drinks. We talked about Snapple and you talked about--

MS. G: [Interposing] Pepsi.

MR. INTERVIEWER: --vitamin water and Pepsi. And let me ask you this, C. You said--I think--did you say something like you felt addicted to it? Yeah. Was it hard to get off of that?

MS. C: It was 'cause I tried replacing it with Coke.

MR. INTERVIEWER: Yeah.

MS. C: But--

MR. INTERVIEWER: [Interposing] With Coke. You were drinking a Pepsi so you tried to replace it with Coke.

MS. C: But it was--I started to get more--after I drink a lot of--'cause I would get--no, I didn't have the - - Pepsis.

MR. INTERVIEWER: Yeah.

MS. C: So I would--after that I would get dehydrated and I just wanted more and more and then I was like no I can't. And then I replace it with Coke. That made it better. It was just….

MR. INTERVIEWER: So now you drink Coke instead of Pepsi.

MS. C: No, I don't drink soda at all now.

MR. INTERVIEWER: Right. So when you went from the soda--when you stopped drinking the soda, was it difficult?

MS. C: It was hard.

MR. INTERVIEWER: Uh-huh.

MS. C: 'Cause everyone around used to have their soda and--

MR. INTERVIEWER: [Interposing] 'Cause everyone around you had soda.

MS. C: So it was hard but…. The juices, I don't know. I like the Snapple. It's all right.

MR. INTERVIEWER: Okay. Anybody else about soda? Opinions about soda. Do you drink that?

MS. G: I never liked soda.

MR. INTERVIEWER: I'll get right to you - - . Hold that thought.

MS. G: In the wintertime I never drink soda. For some reason I can't stand it. When the summertime comes I get that urge for a soda.

MR. INTERVIEWER: Mm-Hmm.

MS. G: I don't drink that much, but I like the root beer, A&W, you know.

MR. INTERVIEWER: Okay.

MS. G: And that's about it.

MR. INTERVIEWER: You like the root beer. What is it about root beer, A&W.?

MS. G: I don't know. I just like it.

MS. D: I like root beer too.

MS. G: I like it with Butter Pecan ice cream. Oh, no. I make the milkshakes.

MR. INTERVIEWER: Sounds good actually to me.

MS. D: Yeah, it's my favorite.

MR. INTERVIEWER: But the root beer, so you like root beer. And is there anything--so but I'll get to you, D, in a second, but B., you've been waiting patiently. Freedom of choice. What do you mean by that?

MR. B.: Yeah. You have a choice to pick what we drink or what we eat.

MS. G: Yes, absolutE..

MR. S: AbsolutE..

MR. INTERVIEWER: This is America, right?

MR. B.: Yes. Here's the thing, if you know it's bad for you or if you don't know it's bad for you, would it be helpful if you check with your physician - - ? I don't know what this means. I don't know what that means. Can you please explain this to me?

MR. INTERVIEWER: That's a good idea actually, S, right. If it's - - concern--

MR. S: [Interposing] Yes. The same--

MR. INTERVIEWER: -- - - .

MR. S: Yeah. There you go.

MR. B.: Then you bring back to him what he said. So if you do that, you go on the right track.

MR. INTERVIEWER: Right.

MR. B.: But if you refuse to--

MR. INTERVIEWER: [Interposing] Yeah.

MR. B.: --you're accepting--

MR. INTERVIEWER: [Interposing] Bad health.

MR. B.: Yes. All the monsters that you don't want to you - - because you don't know better.

MR. INTERVIEWER: Right. So again--

MR. B.: [Interposing] You don't want to educate yourself.

MR. INTERVIEWER: You don't want to educate yourself. Again it comes back to this literacy, health literacy--

MR. B.: [Interposing] There you go.

MR. INTERVIEWER: --that's a problem between--

MR. B.: [Interposing] Yes.

MR. INTERVIEWER: --or a challenge--

MR. B.: [Interposing] Making a choice.

MR. INTERVIEWER: But you need that information to know how to make a choice is what you're saying. I just want to make sure I understand.

MS. G: Even though now it's the summer and everything, this is what I've been drinking.

MR. INTERVIEWER: Okay. So--

MS. G: [Interposing] Over anything.

MR. INTERVIEWER: So you used to go from winter to summer you started with soda and now you're saying now it's summer--

MS. G: [Interposing] Yeah.

MR. INTERVIEWER: --again but you're not doing so much root beer.

MS. G: Just drinking water.

MR. INTERVIEWER: Why is that?

MS. G: Water and--'cause the diabetes.

MR. INTERVIEWER: Okay.

MS. G: I'm not sure.

MR. INTERVIEWER: Right.

MS. G: And like they said I have the beginning of it. They're still testing on that. So I am trying - - too much sugar--

MR. INTERVIEWER: [Interposing] Okay.

MS. G: --in my body. It's like you go to certain places, right. And I tell them I want a cup of coffee. Just put one sugar.

MR. INTERVIEWER: Okay.

MS. G: They put three or four, a lot of stores they just put--and then I'm like you know what I'm better off going to a place that--where you can go make your own coffee.

MR. INTERVIEWER: Right.

MS. G: Because no matter what you tell them they just--they don't know if you got diabetes, you know.

MR. INTERVIEWER: So G can I ask you to not leave the microphone for a second, if you don't mind.

MS. G: Oh, sure.

MR. INTERVIEWER: I want to follow up with what you said.

MS. G: Okay.

MR. INTERVIEWER: The root beer. You love the A&W root beer. Summer after summer after summer you went for an A&W root beer. This summer you didn't. You said because of the diabetes.

MS. G: Yeah. And you--no, this was a while back, you know.

MR. INTERVIEWER: Okay. So a while back--

MS. G: [Interposing] Yeah.

MR. INTERVIEWER: --you started to go away from the root beer. Was it hard to do that? That's my question.

MS. G: Not really, no.

MR. INTERVIEWER: Okay. You can get up now if you want - - . What--anybody else about healthy beverages? D, do you have anything to say? Are you studying the--

MS. D: [Interposing] About the beverages?

MR. INTERVIEWER: About the--so the sugar--sweetened beverages. Do you drink them? Have you drank them?

MS. D: I drink Pepsi.

MR. INTERVIEWER: You do drink Pepsi. Okay. So let's talk about that.

MS. D: It's an everyday thing.

MR. INTERVIEWER: Okay. Not an everyday thing.

MS. D: But sometimes I crave them.

MR. INTERVIEWER: Sometimes you crave them.

MS. D: And I know that sugar is not good but it's overuse of stuff, you know. You have to portion whatever you're doing.

MR. INTERVIEWER: All right. So let me talk about the craving and when you drink it. When do you drink it and when do you crave it? And talking about that--

MS. D: [Interposing] Well say for instance I ate something and I went to sleep. Instead of getting up walking around or whatever, I just fell asleep.

MR. INTERVIEWER: Right.

MS. D: When I wake up I need to get that Pepsi so that I can burp.

[Laughter]

MR. INTERVIEWER: So you can burp. Really?

MS. D: You know, it clears my airways or, you know, or whatever is going on in there.

MR. INTERVIEWER: Yeah.

MS. D: But I'm a water drinker.

MR. INTERVIEWER: Okay. So when you sleep--

MS. D: [Interposing] I drink water all the time.

MR. INTERVIEWER: Okay. But you still crave the Pepsi every once in a while.

MS. D: Yeah.

MR. INTERVIEWER: And when you're at home let's say and you're sleeping and you fall asleep and you wake up and you want that Pepsi, it's there for you 'cause you do buy it and you have it in the fridge or something.

MS. D: No.

MR. INTERVIEWER: Oh, so you go out and get it.

MS. D: I go out and get it.

MR. INTERVIEWER: Okay.

MS. D: Or go get an Alka-Seltzer, either or.

MR. INTERVIEWER: Pepsi or Alka-Seltzer, same thing.

MS. G: I think Pepsi's bad for you. I mean if you drink a lot--I had a friend who used to be a doctor, right. He overdid it. I mean he--and he was drinking diet 'cause he was heavy, right. He would drink so much Pepsi. That's all I'd see is bottles of Pepsi in his house all over. Pepsi and a chain smoker with the Cools.

MR. INTERVIEWER: This is a doctor you're saying.

MS. G: Yeah.

MR. INTERVIEWER: Okay.

MS. G: He was a doctor. He would sit there like he--you know, when you have problems or whatever. He had a lot on his mind. He used to sit there. He was a chain smoker, would smoke one cigarette after another. I was like my God, look at that ashtray. You know, he had three open heart--

MR. INTERVIEWER: [Interposing] Surgery.

MS. G: --surgeries, triple bypass or whatever.

MR. INTERVIEWER: Yeah.

MS. G: The last one he didn't make it, and he was my best friend. He just--he had a heart attack, and I guess he asked my brother or whatever. He didn't get his pills on time and he died.

MR. INTERVIEWER: Okay.

MS. G: But that Pepsi, I don't know. A lot of people always told me that it's not good.

MR. INTERVIEWER: Who are some of the people that tell you that?

MS. G: Just people that I know that said Pepsi is no good I mean 'cause it has a lot of gas and it's--I don't know.

MR. INTERVIEWER: You haven't said anything about sugary drinks.

MR. D: I - - .

MR. INTERVIEWER: Do you drink them?

MR. D: Every two days I have one soda.

MR. INTERVIEWER: Every two days you drink one soda.

MR. D: A small can.

MR. INTERVIEWER: Small can. Same time during the day?

MR. D: No, I do it different times.

MR. INTERVIEWER: Different times. And it's always one can.

MR. D: Yeah.

MR. INTERVIEWER: Okay. Anybody who hasn't said anything about--you're--well you obviously don't mind me just--

MS. A.: [Interposing] Well okay. Well I got into a habit of drinking Sprite because I can't drink dark.

MR. INTERVIEWER: Dark. Who mentioned--you mentioned the dark first, right?

MS. M: I did.

MR. INTERVIEWER: You did, M. Yeah.

MS. A.: And since I have liver problems I started and I - - and I conserve water.

MR. INTERVIEWER: Yeah.

MS. A.: So they told me I can't even drink water.

MR. INTERVIEWER: Really?

MS. A.: I got to take ice and--

MR. INTERVIEWER: [Interposing] Oh, is that right?

MS. A.: Yeah.

MR. INTERVIEWER: The doctor told you--

MS. A.: [Interposing] I take ice and, you know, like to get a little bit of my thirst.

MR. INTERVIEWER: Yeah.

MS. A.: So I got into the habit of drinking something light and I passed it on to Sprite because I really don't like water either.

MR. INTERVIEWER: Uh-huh.

MS. A.: But since they told me I can't drink water like that--

MR. INTERVIEWER: [Interposing] Yeah.

MS. A.: --I've been getting the ice and drink--I come outside and I'll drink Sprite. When I go home I do my ice thing.

MR. INTERVIEWER: And the ice thing is just pieces of ice--

MS. A.: [Interposing] Yes.

MR. INTERVIEWER: --in your mouth.

MS. A.: Yeah. I have to put pieces of ice, yeah, 'cause I conserve water, very much of it.

MR. INTERVIEWER: Right.

MS. A.: They had to take six pounds of liquid they took out of my belly, so I can't drink water.

MR. INTERVIEWER: So do you drink--you like Sprite and--

MS. A.: [Interposing] I love Sprite.

MR. INTERVIEWER: You love Sprite.

MS. A.: Because it's the only one that I like.

[Crosstalk]

MR. B.: But you know a substitute for--

MR. INTERVIEWER: [Interposing] Hold on, B.. We'll get to you in a second. D.

MS. D: A substitute for that is Sierra Mist.

MS. A.: Uh-huh.

MR. INTERVIEWER: Is that better?

MS. D: Did you know that? It's better.

MR. INTERVIEWER: Why? Is there--is it diet?

MS. D: It's diet.

MR. INTERVIEWER: Okay.

MR. B.: Also--

MR. INTERVIEWER: [Interposing] B..

MR. B.: --I was thinking 'cause the sugar content - - --

MR. S: [Interposing] - - --

MR. INTERVIEWER: [Interposing] Hold on, S. We--if you don't mind, I'll let you--

MR. S: [Interposing] - - .

MR. INTERVIEWER: Let's let B. say what he's going to say. Go ahead, please.

MR. B.: Is it that the sugar content in the soda that she craves that?

MR. INTERVIEWER: Right. Does it represent the sweetness, is that what--

MR. B.: [Interposing] Yeah.

MS. A.: Yeah, that's the--

MR. B.: [Interposing] The darkness in the Coke is the same thing but just the color.

MR. INTERVIEWER: Right.

MS. A.: The color--

MR. B.: [Interposing] You can take the color away and still have the sugar in it and it's the same thing.

MS. A.: I know, but I can't drink--

MR. INTERVIEWER: [Interposing] So your question is--

MS. A.: --dark and nothing dark.

MR. B.: It's not that it's dark. It's--

MS. A.: [Interposing] I know but you're telling me it's the same thing.

MR. INTERVIEWER: Right. But you're saying this this is your experience, right?

MS. A.: Yeah.

MR. INTERVIEWER: D, I think--were you going to say something?

MS. D: No.

MR. INTERVIEWER: Okay. S was going to say something. You were going to say something. Go on.

MR. S: About darkness.

MR. INTERVIEWER: Yeah.

MR. S: I don't think it has anything to do with dark because sugar is sugar--

MS. D: [Interposing] Sugar is sugar.

MR. S: --when it get in your body. It becomes glucose whether if you take it dark or light because your body does not recognize that this is dark soda and that is light soda.

MS. D: And every body is different.

MR. S: We're talking health, so, you know, you can take this thing red or you can take it pink when it gets in there and--

MR. INTERVIEWER: [Interposing] Right. But you can't argue with the fact that A.--that's how she feels. I mean she--

MR. S: [Interposing] Yeah. No, I'm just saying.

MS. A.: They told me I can't--

MR. S: [Interposing] I'm not arguing.

MS. A.: --drink nothing dark.

MR. INTERVIEWER: Did the doctor tell you that?

MS. A.: Yeah.

MR. INTERVIEWER: Okay.

MS. A.: That that's not good for me.

MR. INTERVIEWER: Okay. All right.

MR. S: But is the sugar good for you?

MR. INTERVIEWER: Sugar is not good for you. And in fact, I'm going to talk about--

MS. A.: [Interposing] Just like - - .

MS. D: I was getting ready to say is it because we have a lack of knowledge when it comes to that, right?

MR. INTERVIEWER: Well you tell me that what you think about. What do you mean when--what is the question you're actually asking?

MS. D: I mean because you're--everybody--sugar is not bad. All right. But the companies that are making our drinks or our food, food and our drinks are not the way it used to be back in the day.

MS. G: Right.

MR. INTERVIEWER: Back in the day like the caveman day or--

MS. D: [Interposing] I'm talking about--

MR. INTERVIEWER: --even in this--

MS. D: [Interposing] I'm talking about in my time--

MR. INTERVIEWER: [Interposing] When you were--

MS. D: --and my mother.

MR. INTERVIEWER: So--

MS. D: [Interposing] My mother's time.

MS. G: Yeah.

MS. D: My grandmother's time.

[Crosstalk]

MS. D: I'm talking about 1900.

MR. INTERVIEWER: Stop. You know where the bathroom is, B., is that right?

MR. B.: Yeah, yeah.

MR. INTERVIEWER: Okay.

MS. D: I'm talking about the 1900s.

MR. INTERVIEWER: 1900s. Okay.

MS. D: Okay. Man has gotten a lot of knowledge. They're starting to do a lot of new things. We're becoming overpopulated in certain areas. Everybody has different backgrounds, different cultures, things like that or what happening with the land.

MS. G: Changes.

MS. D: The land is changing. They are putting stuff in stuff, nothing is natural.

MR. INTERVIEWER: All right.

MS. D: They talk about what's organic, what's not.

MR. INTERVIEWER: Right.

MS. D: Brown sugar, dark sugar. Like you said, sugar is sugar.

MR. INTERVIEWER: Right.

MS. D: All right. There's cane sugar, there's raw sugar, there's--you know what I'm saying?

MR. INTERVIEWER: Yes.

MS. D: So the companies I believe these drinks that are being made produces jobs, produces--you know. It's just a--

MS. A.: [Interposing] A money thing.

MS. D: It's a thing going on. But we have to learn to say no, read the products. And just like, you know, even when they come down to water that we drink.

MR. INTERVIEWER: Right.

MS. D: Okay. They said New York City water is excellent. I'm not saying that it's not. I think it tastes delicious. But it's with chlorine, right. But then there's spring water. Then there's water with fruits in it. I mean what could I say.

MR. INTERVIEWER: You said a lot of stuff just now.

MS. D: Yes.

MR. INTERVIEWER: I don't even know where to begin.

MS. D: Me too.

MR. INTERVIEWER: - - .

MS. D: We're supposed to be talking about--

MS. G: [Interposing] Right.

MS. D: I said we're supposed to be talking about beverages, right?

MR. INTERVIEWER: Yes. Here, I want to--you said a lot of stuff. I want to talk about one of the things you said and that is--I'd like to hear more about one of the things you said. And that is this issue of when you were a girl I think you said. When you were younger, right, back in the day--

MS. D: [Interposing] Yeah. I'm still nine.

MR. INTERVIEWER: You're still nine. But when you were five--when you were half as old as you are now, you said back in the day. What's different between back in the day--

MS. D: [Interposing] Things were more natural.

MR. INTERVIEWER: What do you mean? So they're more artificial now do you think?

MS. D: More artificial now.

MR. INTERVIEWER: What about drinks and--

MS. G: [Interposing] Of course.

MS. D: Who?

MR. INTERVIEWER: Drinks. How are drinks different? Just more natural?

MS. D: Oh. Say for instance, we used to have Kool-Aid.

MR. INTERVIEWER: Okay.

MS. D: You know, we used to make lemonade.

MR. INTERVIEWER: Yeah.

MS. D: There wasn't a problem of people dying of sugar, diabetes.

MR. INTERVIEWER: Yeah. So--

MS. D: [Interposing] You know?

MR. INTERVIEWER: Okay.

MS. D: Just like you see people with canes and walkers.

MS. A.: There's a lot of chemicals in stuff today.

MS. D: Yeah.

MR. INTERVIEWER: Okay. Yeah. A., you were--

MS. A.: [Interposing] I think they put steroids on the food because I see kids--

MR. INTERVIEWER: [Interposing] Yeah.

MS. A.: --growing up so big and they're only ten years old and they're huge.

MR. INTERVIEWER: Okay.

MS. A.: You understand?

MR. INTERVIEWER: What else about this--

MS. A.: [Interposing] - - something.

MR. INTERVIEWER: Yeah. Okay.

MR. S: And then they have these--

MS. A.: [Interposing] And I've never seen a chicken this big. He said that's not a chicken.

MR. INTERVIEWER: Okay. So that gets back to--I think G was saying this too. So the hormones of--they put the hormones in the--

MS. A.: [Interposing] Yeah.

MS. G: Yeah.

MR. INTERVIEWER: -- - - . So while you were talking about artificial ingredients, preservatives, that kind of stuff, right. There's more of that now.

MS. A.: Yes.

MS. D: Yes.

MR. INTERVIEWER: Is that what you're saying?

MS. A.: Yes. That's--you make more money.

MR. INTERVIEWER: And to make more money. You said the money thing. I think someone else might have said something about--

MS. D: [Interposing] Yeah. I said it.

MR. INTERVIEWER: You said it. Yeah. Okay. Great. So I want to get--tell you again we're here to talk about health - - from a health perspective, public health. And I know--all of you seem interested in learning more about it. There is more and more research that indicates that sugar is bad for your health, right. We all know that. But even more research is coming out now. So the doctors all over the country, all over the world and right here at Union Community Health Center are concerned because they see patients, children who are drinking sodas and who also have diabetes--

MS. A.: [Interposing] Yes.

MR. INTERVIEWER: --or childhood obesity. They're concerned about that. The question is what do we do about it? Right. What do we do about this issue? Some people--you know, they want to get people to drink less soda. What do you think about--I mean again we're seeing health problems among children and there's research that shows it's connected to sugar. What do you think about getting people to drink less soda?

MS. A.: Yeah. They should--

MR. INTERVIEWER: [Interposing] You talked about laws.

MS. A.: Yeah. And I agree.

MR. INTERVIEWER: What do you think?

MS. A.: I was just going to say that. They should have a law, you know, like this is natural, you know, let it be real because they can say it's not true and it isn't, you know. So they should have a law--

MR. INTERVIEWER: [Interposing] Oh, it says--yeah, don't say it's natural when--yeah.

MS. A.: And it's not.

MR. INTERVIEWER: Right. So those kinds of things. Laws about information. You're talking about information it should be--

MS. A.: [Interposing] Yes.

MR. INTERVIEWER: Okay. So what about the idea that more than just information--in public health we talk about--we study this fact about information for people, getting people information, telling people about that sugar is bad for you. But then there's the environment around you, changing the environment. For example, did you notice maybe that the vending machines, there's a machine in the front area that had soda and you seEd to notice that, C. Did you notice the day that they took out soda and now there's no more soda? It's diet soda and water.

MS. A.: Yeah. They made it a big thing about the soda cut down.

MR. INTERVIEWER: Right.

MS. A.: And McDonalds and all that.

MR. INTERVIEWER: Yeah.

MR. D: They make more money after they stop the big ones.

MR. INTERVIEWER: They stop the big ones so they can make more money.

MR. D: - - .

MS. A.: Yeah, so you do that twice.

MR. INTERVIEWER: C, you're nodding your head when I said do you rEmber the soda. You seem to rEmber the day they took soda out of here.

MS. C: Yeah.

MR. INTERVIEWER: How did you--what were your thoughts about that?

MS. C: That's good to me.

MR. INTERVIEWER: That's good to you.

MS. C: Yeah.

MR. INTERVIEWER: Okay. What do you think about it if a place like this, a hospital or a health center like Union Community Health Center said, you know what, no more soda in our vending machines? And in fact, let's not--yeah, go ahead.

MR. B.: That would be a beautiful thing. But here's the thing. People got to educate themselves and a lot of people refuse to do that. They take things for granted. If I have one today, it's not going to hurt me. And then I can have another one two days after. But the time limit it takes to break down that sugar in your body they don't know the concepts.

MR. INTERVIEWER: More people need to understand what--

MR. B.: [Interposing] Understand--

MR. INTERVIEWER: -- - - . Yeah.

MR. B.: --what it does to you.

MR. INTERVIEWER: Right, right.

MR. B.: Instead of just saying oh it takes - - . Give it some time. It's going to wash away. It does not.

MR. INTERVIEWER: Right.

MR. B.: It will do the damage--

MR. INTERVIEWER: [Interposing] Right.

MR. B.: --before it - - .

MR. INTERVIEWER: But I'm not saying give it some time and wash it away. Let me be clear about what I'm saying. Some doctors are saying that places like this all over the country we need to be stricter about--we got to do more than taking them out of the vending machines.

MS. A.: AbsolutE..

MR. INTERVIEWER: You agree. Maybe we should say--

MS. A.: [Interposing] Yeah.

MR. INTERVIEWER: --you know, when patients come in we tell them, you know, this is bad for you if you--

MS. A.: [Interposing] Or put some signs up.

MR. INTERVIEWER: That maybe say what? What do the signs say?

MS. A.: You know--

MR. S: [Interposing] Explain the--

MR. INTERVIEWER: [Interposing] Well let me--let A. continue what she was going to say, please.

MS. A.: You know, well this is a healthy beverage, you know.

MR. INTERVIEWER: Healthy beverage zone or something like that--

MS. A.: [Interposing] Right.

MR. INTERVIEWER: --right--or area that says--

MS. A.: [Interposing] Right.

MR. INTERVIEWER: --you know, please--we only have--only have healthy beverages only.

MS. A.: Right. That sounds good.

MR. INTERVIEWER: That sounds good. Does that sound good to you?

MR. B.: That's a beautiful thing, but what do you do with the supermarket? What do you with the - - ? What do you do then?

MR. INTERVIEWER: What do you do?

MR. B.: It's open season.

MS. G: Like cigarettes. You just burn all the crops--

MR. B.: [Interposing] Yeah.

MR. INTERVIEWER: Yeah. Hold on, G. We'll get back to you in a second. Yes.

MR. B.: But - - kids destroying themselves at the age of 12 through 20.

MR. INTERVIEWER: Yeah.

MR. B.: The damage is done there.

MR. INTERVIEWER: So eliminating it saying this is a healthy beverage zone here. That's your idea.

MS. A.: Yeah.

MR. INTERVIEWER: You're saying, you know, this is just such a small area.

MR. B.: Right.

MR. INTERVIEWER: What about the community and everything?

MR. B.: Yeah.

MS. A.: It has to be the--

[Crosstalk]

MR. D: - - sugar that we have.

MR. INTERVIEWER: Hold on, D. - - .

MR. S: How do you control that?

MR. INTERVIEWER: How do you control that?

MR. B.: I have no idea.

MR. S: We are the ones.

MR. INTERVIEWER: - - .

MR. S: We are the ones--

MR. INTERVIEWER: [Interposing] Right.

MR. S: --who could put a stop to it.

MS. A.: That has to learn how to control it. Yeah.

MR. INTERVIEWER: Okay. So D, hold on. What do you mean? Say what you were going to say?

MS. A.: We're the ones that has to learn how to control that.

MR. INTERVIEWER: Right, right.

MS. A.: You know. But if there are more signs up and if the president was to put his foot down and say--

MR. INTERVIEWER: [Interposing] Yeah.

MS. A.: --well there is no more soda selling in the hospitals or whatever--

MR. INTERVIEWER: [Interposing] Right.

MS. A.: --you know.

MR. INTERVIEWER: Right. Then you think what? That's a good thing.

MS. A.: Yes.

MR. INTERVIEWER: Okay. D, what were you going to say?

MR. D: Yeah. I said you can't eliminate sugar. - - sugar - - diabetes - - .

[Ringing Phone]

MR. INTERVIEWER: Whose phone is that? You?

MS. G: Yes. It always does this. - - .

[Crosstalk]

MR. INTERVIEWER: Your ring tone is sounding like - - .

MS. G: It's mine. It always does that.

[Crosstalk]

MR. INTERVIEWER: All right. Everybody, let's--we're almost--we're getting closer to the end. You guys are having an okay time here?

MS. A.: Yeah.

[Crosstalk]

MS. A.: Good, good. I enjoy it.

MR. B.: It's beautiful.

MR. INTERVIEWER: So--

MS. G: [Interposing] It's a beautiful thing.

MR. INTERVIEWER: So D, so you're concerned that maybe, you know, we don't want to restrict too much sugar but--

MR. D: [Interposing] Yeah. That - - sugar has to go down and they have to - - .

MR. INTERVIEWER: Okay. So - - .

MS. A.: But regardless of what? They're going to still - - , you know.

MR. INTERVIEWER: Yeah.

MS. A.: But it's up to you.

MR. S: Candy, this, that.

MS. A.: Yeah, that's it.

MR. INTERVIEWER: Okay. Back to the question I was asking. S, I'm going to put you on the spot. I'm just going to ask you point blank. What if we had A.'s idea? Sign said sorry no--please don't drink soda in this building. What do you think about that idea?

MR. S: I think it shouldn't be so prompt like it should have an explanation and then, you know, because people need to be educated. When you go to McDonalds there's no such thing that they educate you. Adults need to learn, even if you go to your doctor that it would have some information from your doctor and tell you okay well this is not good for you so that you can bring it to your children.

MR. INTERVIEWER: Mm-Hmm.

MR. S: And they can be educated to because they eat more like Burger King and McDonalds so at least they get information from home because they don't get it outside there.

MR. INTERVIEWER: Right.

MR. S: And that they can have some sense of being to know what the repercussions are, what they can do, what they can--you know, the sugary stuff, you know.

MS. A.: What's going to happen?

MR. S: You know, and the danger of it and how to, you know, to take the relevant precautions, you know, that okay well I can have this.

MR. INTERVIEWER: Yeah, yeah.

MR. S: But it can be in this kind of way. They have water that there's fruit flavored.

MR. INTERVIEWER: Ah, alternatives. You have to have alternative choices because--

MR. S: [Interposing] So you know, because--

[Crosstalk]

MR. S: Because that can be--so like I said, it can be so hard you can get stressed out.

MR. INTERVIEWER: Let me summarize. Let me see if I understand what you said. One of the things you said is you can't have a sign--shouldn't have a sign that says you can't drink soda in here because you have to explain why, right? Is that one of the things you said?

MR. S: Yeah.

MR. INTERVIEWER: Now the other thing I heard you say is you can't just say you can't drink soda here and even because it's bad for you without saying there are other things--

MR. S: [Interposing] The pros and cons.

MR. INTERVIEWER: --the pros and cons but also here's some alternative things, right. You're talking about the--

MR. S: [Interposing] Yeah, absolutE..

MR. INTERVIEWER: So that's what you said - - . Okay. Anyone else before we move on to get to the very end, thoughts about the idea of simply saying at a place--'cause again I'm saying the doctors here are very--not just here but doctors all over the country are very frustrated with the illness that they're seeing in patients and especially children who are obese and who--I mean it's one thing to be--you know, have health problems when you're 50 or 40 or, you know--

MS. A.: [Interposing] But they're coming up.

MR. INTERVIEWER: They're coming up, yeah. And doctors are concerned. So they're saying we should do this stuff. So final thoughts before I get into some other final questions, before I get to the last question. Any last thoughts on that idea of--

MS. G: [Interposing] Just come up with a substitute for, you know, something different but something healthy.

MR. INTERVIEWER: Yes.

MS. M: The president--

MS. G: [Interposing] I'm sorry.

MR. INTERVIEWER: No. M, just let me let G say a little bit more. Tell me more about what you just said if you could. So I asked the question what about just saying no soda here and you said you have to have an alternative--

MS. G: [Interposing] Yeah. Just come up with a substitute but something healthy, something that's going to draw their attention like for kids, you know.

MR. INTERVIEWER: Why do you say that?

MS. G: I don't know, 'cause kids look for sweets, you know, so it has to be something that's going to catch their attention.

MR. INTERVIEWER: It's not good enough if you tell a kid you can't do it.

MS. G: Yeah.

MR. INTERVIEWER: You have to give them something else.

MS. M: Yes, definitE..

MR. INTERVIEWER: Why do you say yes definitE.?

MS. M: Because the young kids today--you know, I don't think they're hardheaded but they have to experience something before they listen and learn. You can't just tell them--they're not the type of generation you can just say listen if you do this, this is going to happen. They got to experience it before they really listen.

MS. A.: It's about the money because they don't care about what's in it.

MR. S: Yeah.

MR. INTERVIEWER: They don't care about what's in it.

MS. A.: As long as they make the money--

MR. INTERVIEWER: [Interposing] They don't care if it's healthy.

MS. A.: --it doesn't matter.

MR. INTERVIEWER: Yeah. So--

MS. M: [Interposing] I'm sorry.

MR. INTERVIEWER: --one last thought from M and then I'm going to get to the end part of our session today. Yes.

MS. M: For instance, years ago the black communities had at--and the Spanish community--anyway. We had a thing about the signs where they had all signs about liquor, you know, where you can go and get liquor and all of that. Now we've advocated it so much and we went against it that you can't find a sign--you may have noticed--you can't find a sign now in our neighborhoods and the - - neighborhoods, whatever, you know, our neighborhoods--that have a liquor, you know, with the lady with the bottle with the bikini on and tell you with an arrow where to go to the next--they're no longer in our community. A lot of people--

MR. INTERVIEWER: [Interposing] - - --please, keep going.

MS. M: We went against that and it happened, so why can't we do something like that about the healthier drinks?

MR. INTERVIEWER: So people in the community made those changes.

MS. M: Oh, yes. We made those changes. They cannot put a sign about a liquor store--

MR. INTERVIEWER: [Interposing] Right.

MS. M: --up in our neighborhood.

MR. INTERVIEWER: It takes communities to get together to do this. - - --

MS. M: [Interposing] And it was only in our neighborhood. It wasn't in any other neighborhoods.

MR. INTERVIEWER: So - - are well managed. Now here's what we're going to do now. The final stage of this. What we're going to do--so again, I told you. Union Community Health Center is concerned about all of this.

MS. M: Oh. Okay.

MR. INTERVIEWER: And you're concerned. You shared that concern.

MS. M: I am too.

MR. INTERVIEWER: We're concerned about our future, right, C, the future children that we have amongst us today. And so we want to study ways to--in advertising, like rEmber I told you about social marketing.

MS. M: Yes.

# CITY UNIVERSITY OF NEW YORK

**Union Health Center Focus Group #3**

**Thursday, June 4, 2015**

**4:30 p.m.-7 p.m.**

Ubiqus/Nation-Wide Reporting & Convention Coverage

22 Cortlandt Street, Suite 802 - New York, NY 10007

Phone: 212-346-6666 ⬩ Fax: 888-412-3655

# Union Health Center Focus Group #3

So I'd like to start by asking you, which is what do you think when you think of the word health. A lot of associations with health. What are your associations?

T.: Teeth.

MR. INTERVIEWER: Teeth. It's the first - - teeth. Are you involved in dental care?

T.: Yes.

MR. INTERVIEWER: Okay, that's often the case in life, isn't it, we see the world through our own experience, and you see teeth all day long and you think teeth. Anything about teeth that is important to you or why you say that besides the fact that you see teeth - -

T.: - - speaking of a healthier point of view, you can tell when a person has a bad diet by looking at their teeth, their gums. You can tell if they smoke, if they don't brush properly or even brush at all. You can even tell bone loss and it's all associated with just a smile, just hi, how are you doing. Ooh.

MR. INTERVIEWER: Yeah, and you see that.

T.: Yes, we see it. He also could agree.

MR. INTERVIEWER: You are also in dental.

MALE VOICE: Yes.

MR. INTERVIEWER: But back to the first -- back to the question. Health; what do you think of?

MALE VOICE: Well being. Being alive.

MR. INTERVIEWER: Being alive.

MALE VOICE: Staying alive, yeah.

MR. INTERVIEWER: Yeah. So really, when I say health, you think about just being alive.

MALE VOICE: Yeah.

T.: Being in shape, exercise.

MR. INTERVIEWER: Being in shape and exercise, yeah. So some of those--some of the lifestyle stuff.

MALE VOICE: Be good also mentally, physically. That is health.

MR. INTERVIEWER: That's health. So why is health important? I mean, I know it sounds like an obvious question, but sometimes we don't always prioritize health and life, right, so sometimes you see things - - . So in public health and people who work - - sometimes they have to go out of their way to explain or to argue for health, right. So why is health, would you say, if you were making a case for health?

T.: Health is wealth, like everybody says, and it's very important because if you have migraines all the time then you can't focus on what you need to do on a daily basis, and you can't even function, you can't even work. So you need to have your health and checking of it to do what you're supposed to do.

MR. INTERVIEWER: Why is it important, why is health important? Don't tell me you don't think it's important, do you?

MALE VOICE: Of course.

MR. INTERVIEWER: Why? Why do you say of course?

MALE VOICE: Well, like I said - - alive, keep yourself moving. But if you're not someone who's sick, basically they - - they can handle on their own. If they have to go through a process, going through the hospital and everything and it takes a toll, and that gives the person - - the family.

MR. INTERVIEWER: So health helps you achieve other things in life, the things that you need to do, basically.

MALE VOICE: If you don't have health, basically you will die.

MALE VOICE: You won't be alive.

MR. INTERVIEWER: So just to stay alive you need health.

T.: But I think a lot of people think--just like you said, mentally, a lot of people think that health is just physical, but it also--I think it starts mainly from your mental stability. Because if your mental stability is not where it's supposed to be, then your health physically and inside, which is - - because personally I have a sister and her mental health wasn't where it was supposed to be, but she was normal to begin with, but her mental illness just kind of went down the drain, so from there she just kind of not took care of herself, so then it led to her habit onto diabetes and just a bunch of other different health issues that now it's physical as opposed to just mental stability. So it starts from mental first.

MALE VOICE: Yes, mental health indirectly affects your body.

MR. INTERVIEWER: That's exactly what you were saying, T., right. It starts with mental and - - maybe the physical stuff wasn't there in the beginning, but it started getting physically deteriorating. But started with the mental. So with the eight people that we had today, the patients, there's some people who can get away with not saying anything. When there's only four people, I think I can actually make sure that everyone says something. So go on. Why is health important?

D.: I'll be honest with you.

MR. INTERVIEWER: Yeah, I'm going to move this just a little bit over here. Now we'll really have problems. Okay, hold your thought there. I want to make sure we get everybody. Sorry, I'm causing all kind of havoc here. Get this here. So you were saying?

D.: I'm going to say it depends on what your lifetime goal is. There is a perfect example, a person that is 35 years old, is not educated, has a mediocre job $13 an hour. Their attitude in life is, "If I die tomorrow, I'll be happy because I don't have to do this anymore," as opposed to a person that has a little better position in life. You want to take care of yourself, because you want to see your retirEnt. You want to move to Florida in the West Keys and retire. So when you say health, it depends on where you want to be. Some people don't care if they live 'til 50. Some people want to live to 80. It just depends on the person.

MR. INTERVIEWER: And the people that don't care don't want to live. You're saying you know these people, you see these people.

D.: I see them. I know some.

MR. INTERVIEWER: Right. What is it about--I mean, is it--what is it about them that--

D.: Their stature of life. For example, a person that's on Medicaid, they don't work. They don't work because they don't want to work, or they don't work because they can't work. Either which way, what's their plan for the future? None. Another way of looking at it, young girls that have kids at an early age. Can't go to school, no one wants to babysit. What are you going to do when you're 25? So that starts at an early age. Now when they're 45, do they want to live to 50? They've had such a rough patch. By the time they 50, they know they're not looking at any money to retire. The had a job where they were picking up garbage because welfare told them to do it. Why do they want to live? So what are their benefits? Eat what you want, fried food, bad food, don't take care of yourself. Go to the doctor when you're in pain. Your tooth hurts. When you have a headache for more than five days, or you pass out in the street, you turn to narcotics, then the next thing it just snowballs into the point of I don't care.

MR. INTERVIEWER: Wow, that's profound. Tell me you - - agreeing.

T.: Yeah, because it all makes sense, your mental stability, that’s where I think everything starts from - -

MR. INTERVIEWER: You said that earlier. But you also were smiling to what she was saying about this issue of people who are essentially kind of giving up is what you're talking about in life, right?

T.: Right.

MR. INTERVIEWER: Anything--I mean, what's your perspective from like - -

T.: Like basically what she said, like sometimes in the neighborhood, like certain neighborhoods, like in the Bronx neighborhood, you see that a whole lot where especially people would come up to you and they just start ranting about their life and what they've been through and you never even asked them, and then you almost feel bad for them, because they're telling you their stories, their situations in life, and it's like they just don't care at all. And they look at them physically and then you see that, they're deteriorating, just by looking at them physically, you can just tell that nothing looks right, and it's like something happened along the lines that triggered them to be the way they are now, so that's why I agree.

MR. INTERVIEWER: Interesting. You see me nodding. You've seen that, the experience of that both have been saying is people who essentially give up on their - - and my question was why is health important. And you said to live and you see - - but then you said there are some people that it's not important, right? Is that really--can you--

MALE VOICE: Yeah, I mean, I think it's just by working here, the type of clientele that we have here. I mean usually--I mean, I don't know about anybody else, but about myself, I try to go to see my doctor at least every six months, keep everything on track so that I never end up being sick. I've been in this country for over 22 years and I've never been to an Ergency room because I've always taken care of myself. But sometimes we have patients that come here and it's like the last rope. They go there to the - - tooth pain, even kids early age, and you can tell that sometimes they've been eating a lot of sweets, especially soda, and the problem they have no control of it. And as the child grows up, I've seen the mentality keeps growing and then when they get to adult, it's already there. They have all the health issues and they end up coming--it's like a cycle.

MR. INTERVIEWER: So what about that. You mentioned soda beverage and sweet beverages, right. Why are you smiling and laughing?

T.: Because I have a favorite soda, but--

MR. INTERVIEWER: What is it?

T.: It's Sunkist.

MR. INTERVIEWER: I wanted to ask you about what your favorite sodas were. So you like Sunkist.

T.: Yeah, but I'm trying--like I have stopped.

MR. INTERVIEWER: So you don't--it used to be your favorite.

T.: Yeah. I mean, it's still my favorite every now and then, like if I go to the store, that's what I would reach out for, or a Ginger Ale, one of those two.

MR. INTERVIEWER: So did you used to drink more Sunkist?

T.: Yeah, but then I switched to apple juice. That's not healthy either.

MR. INTERVIEWER: Why did you like--what was the deal before you drank Sunkist, how often did you drink it, why?

T.: It just makes sense. I don't know, it was sweet. Pepsi gives you gas and Coke gives you gas. Ginger Ale has a bittery taste a little bit, but still okay, and Sunkist was just sweet and was nice.

MALE VOICE: - - flavor.

MR. INTERVIEWER: It sounds like Goldilocks and the three bears, the bed was too hard, the bed was too soft. Sunkist was just right.

T.: It was just right, yeah.

MR. INTERVIEWER: Right in the sweet spot. So then at some point you stopped deciding you didn't want to drink Sunkist anymore. Why?

T.: That was only because I was going to this store and they would sell you like maybe like rice and chicken and you get a free soda. So I just picked up the Sunkist by default. So I stopped going there, so that's one of the reason why I stopped the Sunkist.

MR. INTERVIEWER: Really.

T.: Because I like to save money, so--

MR. INTERVIEWER: And you saved money as well, okay. Anybody else have soda experiences or--yes, D.. What's your soda

D.: Pepsi or Coke.

MR. INTERVIEWER: Pepsi or Coke. Does it matter between the two?

D.: No.

MALE VOICE: I think it's a difference between. I think one is sweeter than the other.

MR. INTERVIEWER: Which one is sweeter?

MALE VOICE: Huh?

MR. INTERVIEWER: Which one is sweeter?

MALE VOICE: Pepsi.

MR. INTERVIEWER: Pepsi is sweeter.

MALE VOICE: I think Coke is.

T.: No, Pepsi is sweeter.

MR. INTERVIEWER: You think Coke is sweeter?

MALE VOICE: Yeah.

MALE VOICE: Pepsi is sweeter. But the taste is better than the Coke.

MR. INTERVIEWER: Well, that's just your opinion though.

MALE VOICE: I don't know - -

MR. INTERVIEWER: Oh, but you agree? You like Pepsi better.

D.: Yeah, I agree. Pepsi tastes better than Coke.

MALE VOICE: It's like sugar but - -

MALE VOICE: They both are like 32 grams of sugar. I mean the 12 ounce--

MALE VOICE: I think the taste is more--you feel the sweetness - -

T.: I just want the caffeine.

MR. INTERVIEWER: Oh, is that right?

T.: Yeah.

MR. INTERVIEWER: Why don't you just drink black coffee then?

T.: I do, but then I run out of coffee--

MR. INTERVIEWER: You can't ever run out of coffee, there's coffee everywhere.

T.: We don't have a coffee machine in the back, so after ten patients you're like this.

MR. INTERVIEWER: Would you like a coffee machine in the back?

T.: Yeah, actually.

MR. INTERVIEWER: If there was coffee here would you--

T.: Yes.

MR. INTERVIEWER: So if there was coffee here you might be likE. to get coffee.

T.: Instead of soda, yeah.

MR. INTERVIEWER: Instead of soda, okay. And as it is for soda, do you leave the building to get soda? I mean, you like to go down to a certain place that you go to?

T.: For my mental stability I need to work off the store. I need to go a half an hour for the morning.

MR. INTERVIEWER: So a soda--so buying the Pepsi, it's not just the caffeine, it's also like getting out of the building, right. It's associated--do you go like a certain time of every day, like every morning at 10 o'clock and you--

T.: No, we usually get our lunch break--our department gets a break from 12:30 to 1:30, so there's--I'm not going to say okay, one whole month I'll get maybe two cans of soda in one month. Which his not like I'm dying for it, but then sometimes I'll go to--just a preference, if I'm in a restaurant and they have more like sweetened beverages, I'll try to stick to something like carbonated water that doesn't have. But if they don't have it, then I'm like okay, well, do you have--well, we have Mountain Dew. Is it a fountain? Yeah. Too sweet. How about--so sometimes the only option you have is soda, a Pepsi or a Coke.

MALE VOICE: I don't know if - -

MR. INTERVIEWER: So M. and R., do you guys drink soda at all, or--

MALE VOICE: Once in a while. I try to avoid because I have like kidney problems, a long time ago, so I try to avoid that.

MR. INTERVIEWER: Is it hard to avoid it?

MALE VOICE: Huh?

MR. INTERVIEWER: You say you try to avoid it. Like would you really want to be drinking it?

MALE VOICE: I really--I'd rather have Pepsi than have water.

MR. INTERVIEWER: You're acting like that's a funny thing.

D.: Yes.

MR. INTERVIEWER: It does not make sense, right.

MALE VOICE: That's what I feel, but I try because I'm taking care of my health. I know that soda is not good for me for many reasons. So--

MR. INTERVIEWER: But there's a lot of things that aren't good for you, so we can't do everything perfect.

MALE VOICE: But once in a while I take soda.

MR. INTERVIEWER: Pepsi is what you like, that's your favorite one?

MALE VOICE: Yeah.

MR. INTERVIEWER: And you like it better than Coke.

MALE VOICE: Huh?

MR. INTERVIEWER: You have made it very clear tonight that you like Pepsi better than Coke.

D.: May I offer you a beverage?

MR. INTERVIEWER: Those Pepsis and Cokes up there have been there--were up there a while, so they may be stale anyway. But if you want some, you're more than welcome to have them. If you'd like some - - you like Pepsi, and I forgot what you said.

D.: Coke.

MR. INTERVIEWER: You like Coke. What about you?

MALE VOICE: Pepsi.

MR. INTERVIEWER: You like Pepsi. How often--do you drink it a lot?

MALE VOICE: Maybe about twice a month.

MR. INTERVIEWER: Who else said twice--you said twice a month. What is this twice a month thing? Really--

MALE VOICE: Usually I have - - when I go out - - to eat pizza, and according to her pizza goes with Pepsi.

MALE VOICE: Yes, pizza without any soda, it's not the same.

MR. INTERVIEWER: It's not the same, yeah.

MALE VOICE: Yeah, usually I drink it whenever I have pizza.

T.: It's like wings and beer.

MR. INTERVIEWER: Wings and beer?

T.: Yeah, like wings and beer.

MR. INTERVIEWER: People drink pizza and beer, though, but not pizza and beer, no?

MALE VOICE: No.

D.: Oh, yeah, beer goes with everything.

MR. INTERVIEWER: Okay, well, we're not going to talk about beer. But actually we are here to talk about soda and sweetened beverages, energy drinks, soda. They're sweet, and you work in the dental unit. And there's increasing research that shows that these beverages are actually causing--that we all know about--

MALE VOICE: Diabetes.

MR. INTERVIEWER: --about--

MALE VOICE: Obesity.

MR. INTERVIEWER: We know about obesity, we know about diabetes, and you know about teeth. But there's more--other things, too, that we're learning as public health and medical researchers relating - - to heart disease, cancer, stuff like that. So the physicians upstairs are involved in--and all around the country, actually, are getting more and more concerned about this, and so they're saying to themselves, we are seeing more children with diabetes, we are seeing more children with tooth decay, we are seeing more children that are obese, and there's some other research that connects soda specifically to these conditions. So the physicians say we are really getting sick and tired of this. We want to do some things about it.

So for example, just trying to get people to drink less soda. So we've all had a good time talking about it, so - - . What do you think about that, do you think it's important to get people to drink less? Do you think it's important to get kids to drink less?

MALE VOICE: Yeah, absolutE..

MR. INTERVIEWER: Yes, absolutE., yes, definitE.. What does that mean, it's important? I mean, what would you do to do that? How would you get--I mean--

D.: Mandate.

MR. INTERVIEWER: Mandate. What do you mean by that?

D.: Sorry--

MR. INTERVIEWER: Can I get you a water?

D.: No, banned it, like McDonald's had this whole you can't buy no more than 20 ounces, so what the people would do, they'll get two. You can't buy--

MR. INTERVIEWER: Actually it wasn't McDonald's--excuse me - - . It was the mayor of New York, actually.

D.: Right, they started with these regulations.

MR. INTERVIEWER: Right, and what do you think about those regulations?

D.: I say they're not firm enough.

MR. INTERVIEWER: They're not firm enough, okay. M. you were going to say something.

M.: That many places you get free refills.

MR. INTERVIEWER: So what about that free refill, you think they shouldn't allow free refills?

M.: I think they should control that more.

MR. INTERVIEWER: This is America. You're saying business can't--we want to do free refills, the customers like free refills, you're saying no?

M.: It's something that indirectly affects the health of the people. Sometimes people in general doesn't know the consequences. I don't know, like in Facebook I think I saw something about the Pepsi and since that I tried to stop drinking that.

MR. INTERVIEWER: I'm sorry, what did you see on Facebook?

M.: Like a little--

[Crosstalk}

M.: No, no, that when you--

MALE VOICE: - -

MR. INTERVIEWER: Oh, that, right.

MALE VOICE: When you--

MALE VOICE: - -

MR. INTERVIEWER: What is the deal? I mean, you boil--what happens?

R.: It becomes like a grease, like--

MR. INTERVIEWER: A piece of meat.

R.: Yeah, like - - something like that.

MR. INTERVIEWER: - - disgusting when you - -

D.: I saw one that was a piece of raw meat, a piece of beef. The put the beef in a pan and threw soda on top of it and left it for 24 hours. Worms started coming out of the meat, and it was caused by the soda.

MR. INTERVIEWER: Yes, T., please.

T.: Okay, so I'm actually going to go the opposite direction in terms of banning and--

MR. INTERVIEWER: Hold on a second, we just want to make sure we - - go ahead.

T.: So I'm going the opposite direction in terms of banning because the way America works, we're in a capitalist society, so every business wants to make money, they want to have a turnover no matter what. So it doesn't matter what the mayor does, try to ban this, there's always going to be another mayor that's going to come in office and take off that ban. So it really would not even make any sense. I think it all starts with having self control and learning that at home. That's your own personal responsibility of what you're going to take and put in your body. It's not somebody else's responsibility within power to help you with what you take into your body. So that's what I think.

MR. INTERVIEWER: So this is very common and very well tracked conversation in America, this issue of individual control, individual responsibility and somebody imposing responsibility. And you said that imposing responsibility. Some people say it's wrong to impose it on an individual. You didn't so much say that, I didn't hear that, you say that so much as it's not that it's wrong, it's just that it's not going to work, because--

T.: Yeah, it's just not going to work.

MR. INTERVIEWER: But do you think it's it's--I mean, is it immoral? Because some people think it's immoral for the Government to tell people that they can't drink soda - -

D.: I disagree, because if the Government is going to take my taxes to help Juanita from around the corner that wants to drink a two-liter of soda for breakfast lunch and dinner, I say stop her from drinking it so that my tax money can go into better things in life, like education. So in the sense I guess we are a capitalist country, and yes, you have the--in the Bill of Constitution you are allowed to freedom of speech, pro-choice. But if those decisions are done poorly, then the government as a unit should take control. The same way how they banned fire arms. It was in the Constitution. You have the right to bear arms. Different states, you can't. New York, ten years in prison, right?

Going along that line, not to side track, but if it's such a problem and we are doing this research to see that these beverages do give problems, long-term problems, stop it from now, ban it, that way we don't get to that crossing point.

MR. INTERVIEWER: I want to get back to you, tell me, since you--hold your thought, M., if you could. - - I asked you this question and D. was very passionate about her response, which I appreciate. But I do want to get back to your--my question to you is you said it's not going to work. But - - do you think it's just problematic for a government to do that? Especially from another country. You've seen the world from other countries, right? So you see how--

T.: I don't think that's problematic, but I just don't see it working.

MR. INTERVIEWER: You just don't see it's going to work. You don't--as I said, when I said problematic, like you don't have an issue with it, like you wouldn't--

T.: I don't have an issue if they ban it or if they put a limit on it.

MR. INTERVIEWER: You just roll your eyes and say it's not going to happen.

T.: It's just like it's just not going to happen. But I want to pick it back on what she said about--oh, I'm trying to rEmber now. She said--

MR. INTERVIEWER: She said--well, let's see, what did she say.

T.: It was the last sentence.

MR. INTERVIEWER: Anybody rEmber?

T.: It was your last sentence.

D.: Maybe I'll rEmber. About the government?

T.: It was something, your last sentence.

MR. INTERVIEWER: Oh, well, you started about talking about a hypothetical person on the street.

D.: Right. Okay, now I rEmber, whoo, yeah. Okay, so you were saying that--the way a hospital makes money is by having more patients, right.

MR. INTERVIEWER: More sick people, right.

D.: More sick people. So I'm not trying to sound really ridiculous or mean or anything, but that's how a hospital stays in business. So--

MR. INTERVIEWER: You're not the first person to say this.

D.: Yes, but if you put a limit on certain things, then hospitals is not going to have certain patients.

MR. INTERVIEWER: If everyone is healthy, then a hospital goes out of business.

D.: Exactly, and then we would probably not need that many physicians, and a lot of people would probably not even go to medical school to study those type of things.

MR. INTERVIEWER: Dentists would be--people who work in the dental trade and profession would be out of business, too. So you're shaking your head, like--

D.: No, of course not. Because if you have--first of all, there's people that genetically you have bad teeth. Genetically, you're going to be a diabetic. These are things that you cannot, no matter what diet, exercise you do, if it's going to happen, it's going to happen. Now if you want to maintain yourself healthy, yes, you're going to do what R. does, go to see the doctor every six months. Monitor what you eat, like T.. But if we can focus on something more positive instead of negative, then that's great. Right now all we are focused on, and that's not just us as a hospital, it's just like society, oh, you're obese, you're fat. These are things that people are now starting liposuction. Liposurgeons, off the roof. Prosthetic dentistry, off the roof. Why? Because people want to look a certain way, but they're not going behind the fact that to look that way, maintain it, you need X, Y and Z.

So it's kind of hard to say we'll be out of business because people go through Alzheimer's. You cannot beat that. Heart disease. You can't beat that. And that's just saying--glaucoma, you can't fight that. And of course, everyone is going to have a tooth ache. You're going to always feel like oh, I need to clean, I need this, I need that. In any position you're going to always have a job, it just depends on which way you want to see it. For example, us, people are not very knowledged on dental care. We get Ergency - - . We get Ergency patients that walk in, oh, when was the last time you went to the dentist? Um, about ten years. And you've got four teeth left. Okay. Now if we promote positive care, that won't happen. Also when it comes down to people knowing the right and wrong, like I said, it just depends on where you live and your society. If we get more education going in these places where they don't care and start to care, then they'll know that every six months you have to see a doctor. All doctors. That's something that us--treating patients, when we'd speak to them, they'd say, oh, I didn't know that.

M.: Yeah, but that's--I think that's what people in general needs, more education. If we don't - - that education so people won't know how to stop. For instance, now they--I feel okay, especially in New York because there is a lot of places that are smoke-free areas. For instance, hospitals, restaurants, the subway. And I was on vacation recently. I went to another country. And anywhere, at the subway, restaurants, smoking, smoking, and it affects me.

MR. INTERVIEWER: That's the way it was in this country 20 years ago or whatever, and now it's not like that anymore. So you go to this other country and you're like wait, I notice it, you're different.

M.: Exactly. So it means that a big campaign against tobacco, it's working in New York because they are promoting. So if we do the same thing with the sugar beverage--

MR. INTERVIEWER: Yeah, what about that? What if we do the same thing with sugar beverage?

M. I think we can control a lot of diseases, yes. Of course.

MR. INTERVIEWER: So what do you think about that now? You said a ban. What about--let's talk about - -in the City, you said the mayor--a new mayor would come along and say that's not going to work. - - American public would not - - that. What about a smaller organization, like a health center, like - - or some - - We've been in soda. We are a health center. We are about promoting--not only do we treat disease and treat dental decay, but we also want to promote it. Why wouldn't we promote health, right? So I'm going to ask you like what are some things that can make a healthier promotion - -. What about--well, let me start by that. Let's talk about soda. What can Union Community Health Center do to make a healthier environment here?

M.: Maybe they should start working on the - - . It's horrible.

MR. INTERVIEWER: Now it's changed, I don't know if you know, but they took out soda there. Did you know that?

M.: I actually never bought soda from there. But the last time that I went there to buy a snack, they had those candies there and those snacks. They're just horrible. They're worse than soda.

MR. INTERVIEWER: Vending machines.

M.: The candies that they have there is--and snacks is horrible.

MR. INTERVIEWER: Anything else about Union here, or--'cause you're at St. Barnabas, is that right?

D.: They need a healthier choice because in the cafeteria--I mean, there's juice, but the food that's there, it's not really appealing, and most of them buildings in St. Barnabas, the campus, like there is no vending machines, like in the offices--like on floors, there is no vending machines. So maybe they switch it with something more healthier, then people would not go to the cafeteria as much. But they want people to go there, obviously, to pay.

MR. INTERVIEWER: Yes. So quick thought before I ask this - - specific, but just in general, how to make this Union Community Health Center healthier, than the vending machines - -

T.: Lectures.

MR. INTERVIEWER: Really, give more information.

MALE VOICE: Yes.

MR. INTERVIEWER: And just give people--and lectures, there's a lot of one on one, patients, but if we brought a bunch of paper in one room - -

D.: - - because--sorry, I've got bad allergies.

MR. INTERVIEWER: No, you're saying interesting things, so take care. Take what time you need.

D.: For example, for prenatal they have a group of first-time moms and they teach them how to breast feed, burp, and teach them how to maintain a healthier type of environment for the baby. Like when you feed, don't put them this way. You have someone that, in the hospital, teaches you that. They're the only department that does that. No other medical facility will ever sit with you and teach you things like that. I mean, when it comes to different departments, you do have like a nutritionist and you say the word nutritionist, nutrition. I'm 450 pounds, I'm okay. The mentality again kicks in where--but if they don't--you say free health assessment. A different kind of word that will trigger, so kind of like bamboozle. I'm going to bamboozle you into thinking it's something else, but when you sit here, you're going to know. You're going to walk out of here with knowledge, and you're going to know that your choices need to change.

MR. INTERVIEWER: And do you think the dental with - - would be one thing to do the same kind of thing, right, group - -

D.: AbsolutE., yeah. We have--

T.: Sorry, you can finish. Go ahead.

D.: No, I was just going to say we have more than enough residents, I'll volunteer one.

T.: Okay, so I'm sorry I keep going on like the other side.

MR. INTERVIEWER: That's okay. That's what we like, don't say sorry.

T.: She said for the assessment, but the thing is that--the truth of the matter is people are lazy, and no matter how many brochures or free assessment that you have, sometimes people just do not care. It's just like an extra pile of paper that's just going onto that person. And the other day, St. Barnabas actually had a free assessment where you did blood pressure, you checked--they talked about sleep apnea, they talked about different things. But the campus is big enough, much bigger than Union, but it wasn't a lot of people that were there. But the physicians, nurses were there, but it wasn't a big turnout of people. So it's like you're telling employees, hey, come to this event, but employees will be the function or I think their function is that okay, I'm here to do my job, boom, boom, boom, all right, 9 to 5, I'm going home, I'm leaving.

Okay, so you tell an employee there's something going on at 12:30 or 1 o'clock, oh, there's a free assessment, let's check this, let's do that, most of them would not even go. That's just the way I see it, what I notice just going around campus and stuff.

MR. INTERVIEWER: R.?

R.: I have nothing to say on that.

MR. INTERVIEWER: Okay, I thought you were going to say something.

T.: I think that would be a way to kind of like bring them in. I mean, I'm not here for the $20, but I'm not going to say promote the $20 either. I would say maybe with more time or you tell them some kind of incentive. I'm not going to say physical money, but some kind of incentive where you say, I don't know--

D.: - - did something about a metro card or something.

T.: Right, like you could--

D.: Like unlimited metro card for 30 days.

T.: Like a little metro card, but then again I think about it like this, it's coming out of my tax money, I don't want that. But something along the lines of where maybe here, for example, here, you go from department to department and you tell them hey, if you do this, you'll qualify to win a raffle. But you have to go to all three sessions and you qualify to win a raffle. What's the chances of 15 people showing up for a raffle? A lot more, because A) you actually win more because you only got to give one person a gift instead of giving 15 people a gift. So that's one good thing.

MR. INTERVIEWER: You have good strategic - - (laughter) You'd be good in business. I want to now go back to what you said, M., and you said something about banning soda, I think you said that, and that's something that, again, some of these hospitals and community - - it's just like you and me are talking about, the idea of saying, as much as you all like your soda, no soda, and we really don't want to have soda because it's the kids, it's the moms with their young kids, the pedes upstairs are saying that they see that, they see obesity, they see diabetes. We've got to do something about that. Well, why don't we - - and you talk about the cycle, right, so - - make sure the parents don't come in. And even among the staff and employees, say look, you have to create an environment--and you've touched on this thing. You said if it's there, it's--if you give the information, they don't necessarily read it. So you've got to make it so the environment - -

MALE VOICE: - -

MR. INTERVIEWER: So what about that idea of what if Union Community Health Center said we're not going to have soda here in the building?

T.: People will strike.

MR. INTERVIEWER: People will strike.

D.: Yeah, because there's a--

MR. INTERVIEWER: - -

D.: There's a union at 1199, and they're very passionate, every single 1199 person. They're very passionate about their benefits or whatever is going on, so I know they would be one of the first folks to stand up and say no, you cannot impose such--

MR. INTERVIEWER: You can't take away my sugary, unhealthy beverages. I have a right to that.

T.: They will take it to the union, they'll find a delegate. They'll do all of that.

D.: And then the delegate will sit there and fight it and say guess what--

T.: - -

D.: No, the delegate will sit there and say, well, this is your choice. Either you live 25 years or you die tomorrow, you choose. Again, it's all about the educational portion behind it. A lot of people are uneducated with the facts behind the soda. Perfect example, my last patient, diabetic, high blood pressure. 245 pounds, blood pressure was 199/99, and his pulse was 87. Sir, you're going to catch a stroke. He's like, so, I'm in pain. What has to be done? All his teeth are rotten, have of them have to go, root tips in disaster, disaster. Floating bomb. I tell him, okay, today we're not taking out your tooth because you haven't eaten, #1, and you're diabetic. Do you know that you have to eat before you come here? Oh, I ate at 11. It's 4 o'clock. Yeah, I know, your cholesterol, do you have any problems with your cholesterol? Oh, no. You sure? You're 240 pounds. You really think you don't have cholesterol problems? Last question, he tells me so what's going to happen? I said you're going to get an extraction. You're going to come in Monday morning and you're going to have a healthy snack with you. Mind you, key word, healthy. Like what? Like a protein bar and a water. Why? Because you're going to get up at 7 o'clock in the morning. You're going to take a shower, you're going to brush your teeth. I have to be physical with these people, like very technical. Shower, brush your teeth, have your medication, eat breakfast, come here by 9 o'clock, register, sit down, and wait. According to how many people come, you could be seen from 9 to 12, anywhere in between. As a diabetic, what happens? If you don't have sugar, or if you don't have your body--your glucose on point, you have a problem. It's either going to go up or go down. You don't have your medication. You have to be stable. He didn't know that. Like okay. He's like so after the extraction, what can I eat and can't eat? I said you an eat after four hours if you're not numb. You could drink something. Oh, like a soda?

MR. INTERVIEWER: That's the first thing he said.

D.: First thing out of his mouth. I told him how about carbonated soda with like a flavor. What's that?

MR. INTERVIEWER: Carbonated water you mean.

D.: Right, carbonated water with a little bit of flavor. Does it taste good? I said yeah, it has like lemon, orange, you should try some. He's like, so no Pepsi? I said no Pepsi. You shouldn't be having Pepsi anyway. You kidding me, you want to kill yourself?

MR. INTERVIEWER: So that's a long conversation. You're telling a pretty long story and that's a long conversation.

D.: And that's the truth. That's how technical you have to be.

MR. INTERVIEWER: So wouldn't it be easier if you said--it wouldn't solve the problem, but would it make things easier if we started saying look, we've got to get serious about this. No, it wouldn't. If we said no soda when you walk into Union Community Health Center. Patient, staff, parents, we are going to ask you, please, don't drink soda. What do you think about that idea?

R.: I think it would be good. I personally don't have a problem with that, but I think it would be good. It would be a great idea.

MR. INTERVIEWER: Let's talk before--because I know--we're answering this question two ways. One is how will it go over well. It's going to go over terrible, the union is going to go on strike. And then it's how do you personally feel. So let's--can you talk a little bit about your personal view, like you think it's a good idea. Why?

R.: Personally I think it's a good idea because I don't drink that much soda. I really understand the consequences of soda. Diabetes runs in my family so I'm trying to avoid getting it to me in a early age, so I'm trying to eat healthy and all that. But I think patients and regular people will understand that starting with bad choices early is going to bring later on a sickness fastened to your body.

MR. INTERVIEWER: The - -

R. Yeah.

MR. INTERVIEWER: Tell me, keep answering about it's not going to fly. What about personally. If you went to--you like Sunkist but you don't drink as much as you used to, but - - St. Barnabas, just like you can't smoke, right. What if that happened, how would you feel about that? Not about--

T.: I mean, it's not a big deal, really, it's not.

MR. INTERVIEWER: Okay, just because you don't really drink a lot.

T.: Yeah, I don't drink a lot of it, so it's not like something that I'm--some people are personally attached to certain things. I'm not personally attached to it, so it's not - - anything like that. But some people, they can't just stop the habit. So it's not a habit for me so it doesn't really matter.

MR. INTERVIEWER: But what about the idea that is it a good idea to do it, and again, not so much for you, but just is it a good--would it be a good thing if--somehow we get past the union thing, get past--would it be a good--

T.: It sounds interesting. Would it work? I don't think so.

MR. INTERVIEWER: You don't think so, right, no, thank you for saying that.

R.: That could be if we start promoting that--

M.: Because I think it depends. It depends the level of education that you have. For instance, me--anything that benefits to me, I will accept it. It is like that. It's something that won't--

MR. INTERVIEWER: Please.

D.: But you know, you're going to listen, that's just what I see. Like you said the smoke-free campus on Barnabas, tons of times it says there do not smoke. - - like it says in the meeting, do not smoke on--but you still see people smoking on the campus.

R.: Right under the sign somebody is smoking. You see that at Hunter College.

D.: Yeah, the no smoking smoke-free campus, and you see some people hiding behind the trees, still smoking on the campus. Or they're like two feet away. But there's like a certain feet away that you're supposed to be away from the campus.

T.: 200.

D.: Yeah, but they're like literally two feet away. And if you see the way the security--I don't know how to describe it. There's a security post and they're right next to that security post, and the security post is on the campus. And these are employees that are smoking and they're creating a bad image that contradicts what St. Barnabas is trying to sell to the public. So when you tell people do not drink soda, it's banned, they would take the soda and drink it outside regardless, in front of the hospital.

MR. INTERVIEWER: So you think people would do the same thing, essentially.

T.: Yeah, depending on how attached they are to it.

MR. INTERVIEWER: Right, and they're pretty attached. You talked about you like Pepsi and you don't think it's a problem. You would think it's a good idea. Even though you drink Pepsi, you still think it's okay to ban it.

M.: AbsolutE.. I know it's something that affects me, even though I do it sometimes, but I will accept, yeah, definitE..

MR. INTERVIEWER: Great news, we're getting toward the end. So I want to conclude by playing these videos, but before we show you the video, I wanted to tell you that Union is interested in figuring out ways to get people to drink less soda. They're tired of seeing--as I said, the doctors are really exasperated about seeing so many of these kids. So talk about the idea of - - that's one of the reasons you asked why are we here for this research, and now I'm telling you is that we want to explore this idea. And now you see kind of why folks - - important, because you're giving--all of what you said is more than just I support a ban, yes, or I support--I'm against the ban. You talk about why it's not going to work, even though you drink it, you know what I mean? So that's what we're getting at. Go ahead.

D.: One thing I just want to add to what she said, which I agree.

MR. INTERVIEWER: Okay.

D.: I agree that the rebels, the I'm going to smoke over here, if Union and St. Barnabas is really serious, then there is a thing called corporal punishment. Get written up. I bet you you'll stop. Three write-ups, a suspension. I bet you you won't touch that soda. I bet you you won't smoke. You either pay your bills, keep your job, or take the lush route. It's up to you. And with the patients, because we have patients--we have signs that say no food. They're eating at 8 o'clock in the morning, they come in, the food in their bag. 9:30, they're sitting there, breakfast, fried chicken wings and fried rice. 9:30 in the morning. Miss, you can't eat here. Security won't tell them nothing. They'll walk out, go get a egg roll, go get a cheeseburger, come back with McDonald's and sit there. Security will see them coming in and won't say, "Excuse me, miss, you can't have any food with you. You can't eat here." So I'm with it. However, they have to have more security because--

MR. INTERVIEWER: It needs to be enforced.

D.: --we need to have this the proper way, because the patients always come to the staff and want to kill us.

MR. INTERVIEWER: Oh, really?

D.: Yes. The security guard, by the time--we've been assaulted plenty of times, and trust me, it ain't for a soda. Every time that happens, we have to defend ourselves and kind of stick together like hey, we have to call each other, like literally, from the bathroom. Did you hear that patient? Pay attention. Call security. Hey, M., close your door. So what happens if we have a patient that is a mental patient, they're not mentally stable--

MR. INTERVIEWER: Right, they're sitting there with a soda and you say excuse me.

D.: They're sitting there with a soda--

MR. INTERVIEWER: And you say please don't and they start screaming at you.

D.: --and we tell them, "Hi, Mr. Valez, I'm sorry, but can you put your soda away because this is a soda-free"--

MR. INTERVIEWER: Yeah, building or whatever.

D.: Do you know the words that's gonna come out of that man's mouth if he doesn't grab me by my throat and throw me against the wall?

(Crosstalk)

T.: They're not going to want to come back. And then they will tell--

D.: They'll come back. They'll come back, but they're going to put up a fight. So there has to be some--I know the point is to kind of figure out that medium in between, but if it's going to be done, we have to have one thing for sure, and that's more security because--

MR. INTERVIEWER: EnforcEnt.

D.: More enforcEnt.

MR. INTERVIEWER: Because you can only--you can pass a law, but if you're not going to enforce a law--

D.: There's no point.

MR. INTERVIEWER: --what's the point, right. So you agree with it, you're fine with it.

D.: Yeah.

MR. INTERVIEWER: But it needs to be done well.

T.: And that's a social responsibility that--

MR. INTERVIEWER: Keep going.

T.: That's a social responsibility where the employees actually own this mandate and say okay, this is what the hospital wants, this is the guidelines, and this is what I need to follow.

MR. INTERVIEWER: How do you do that though. Tell me. I want you to--

T.: I mean, many times they have employees sign like hey, this is part of your job description, this is what you're going to do, this is what you need to follow.

MR. INTERVIEWER: How do you get them to really--

T.: You tell them you're going to get a tax break.

MR. INTERVIEWER: You keep coming back to - - taxes. (Crosstalk)

T.: People care about their jobs, literally, so if they - - enough for not following company's procedures and what the company wants--

D.: Corporal punishment.

T.: --then you're not following company procedures. You're not aligning with what the company views for itself.

MR. INTERVIEWER: So strict rules and regulations.

M.: I think one solution--

T.: That's what people can learn, punishment.

M.: --could be like going back to the smoking, the same thing that is happening, increase the price. This is in general. If they increase the price for the soda, the less people are going to consume.

# CITY UNIVERSITY OF NEW YORK

# SCHOOL OF PUBLIC HEALTH

**Union Health Center Focus Group #4**

**Monday, June 8^th^, 2015**

Ubiqus/Nation-Wide Reporting & Convention Coverage

22 Cortlandt Street, Suite 802 - New York, NY 10007

Phone: 212-346-6666 ⬩ Fax: 888-412-3655

# Union Health Center Focus Group #4

[START RECORDING]

G.: [laugh]. Okay. So, just to begin, we are here today - - because, this is a Health Center, as you know, and they want to be healthier. So, we are looking for a way to make this community a healthier one for the patients, for the employees, for everyone. So, just to begin, I’d like to ask –I’m going to ask the question. Whoever wants to answer can raise your hand and can talk, but I want the answer from everyone if you have an opinion about that. So, first, what do you think about when think about health? So, when the word “health” comes to mind, what do you think about? M..

M.: Oh, when they say the word you just said, health, it means a good alimentation, good nutrition, good rest. Because us, as human beings, have the right to rest eight hours in order for our neurons and cerebellum can get the energy they need in order to think. But that also includes good alimentation. How to feed ourselves? Feeding ourselves adequatE., healthy, like, with vegetables, fruits, carbohydrates, proteins that –for example, my plate, which is what they mostly mention at the hospitals, my plate. We can feed ourselves and mainly drink eight glasses of water per day, which is what they mandate for a healthy skin, and for the oxygen and blood to circulate [crosstalk]--

FEMALE VOICE: I think that the most important thing about health is nutrition.

M.: Nutrition, and rest, of course.

FEMALE VOICE: Exactly.

G.: Okay. And J. had--

J.: She already mentioned everything. The only thing needed is exercising a lot.

G.: Okay. Exercising. So, what else comes to mind when you think about the word “health” again? Well, F.

F: Well, the same, she said it all.

G.: M.A..

M.A.: I understand that health means a way to a better life, a higher level for a better living, physically and mentally.

G.: - - yes - - [Background noise]. E., do you have anything to say?

E.: No, I agree with what was said.

G.: You agree with everyone. Okay. Okay. So, let’s go on - -. Anyone else has anything else to say? Well, let’s move on. Another, so, next question is, do you think it is important to be healthy?

J.: J.. DefinitE.. [Background noise].

G.: Why?

J.: Because if you don’t take care of yourself, mentally healthy, as she said, nutrition, everything, no, the body weakens. And it can be the littlest thing, like liquid, water. If you don’t have it in your body, that will cause a big difference. So, and it’s just water.

G.: Uh huh, it can make a difference.

J.: Uh huh, let’s say, a big difference.

G.: Okay.

M.: And yes, part of what the young woman said, yes, because if, if we are lacking water in our body, for example, we know that we will get dehydrated and we will get damaged. Therefore, nutrition very, very, very important, and how to hydrate ourselves. Of course, hydrating ourselves in a healthy way, because we see many people that we think they’re hydrating with a Coca-Cola or whatever, no, we’re getting damaged. Because we’re not hydrating our bodies, we’re poisoning it, intoxicating it. So, we’re not going to have good health. And it’s very important to keep good health in order to be alive, of course.

G.: Of course. Anyone else has an opinion?

F: Not using drugs.

G.: Okay. [Background noise]. [Crosstalk]. [Background noise].

FEMALE VOICE: And taking your medication.

G.: Uh huh. Anyone else? [Background noise]. Who else [crosstalk]? [Background noise].

M.: The baby is saying it now.

G.: Very well. I’ve had everyone’s answer. So, now, we’re going to talk about soda and sugary drinks.

M.: Chang, chang, chang, chang. [Laugh].

G.: What do you think about them?

ALL: Horrible, horrible [crosstalk].

G.: One at a time. One at a time. Mr. F - -.

F: Well, I really know that they are harmful, but I, like the doctor told me here, at this hospital, what I drink, I drink water, but I drink Canada Dry. Instead of drinking - - I drink [crosstalk] because I know it’s harmful. I am aware it is harmful, and I’ve tolerated it with - -.

G.: Okay. J., what do you think about…?

J.: I said horrible, and the example, right now [crosstalk] [laugh] it’s not good, but, as I’ve explained to her that –once a week- that I put - - I used to give them like, how do you say freedom?

G.: Freedom.

J.: Like freedom. I let them drink sodas, juices. And besides that the sugar, which - - diabetes, my grandfather has recently died of diabetes. I don’t want them to get used to that either. And like I said, it’s not the best example, it has helped to teach him because [crosstalk] water is better, and that makes him feel better.

G.: What else? M., what do you think about sugary drinks?--

M.: Well, I think that’s terribly terrible. That’s one of the first factors that damage your health. Why? Because it damages your kidneys. Because you think, it’s really tasteful. At the time, a soda, it’s very good, but we’re not seeing that we are, we are giving, opening the road to diabetes. Even more if we have a descendants, first, second generation in our family, to take them to diabetes. Another one, we damage our kidneys with those artificial colors, preservatives that those drinks have. And the other one is that, uh, damn, it’s gone.

No, the other one is that [crosstalk] yes, that the sugar, as I said, for example, when we have a soda, in a soda we have 10 tablespoons of sugar in our body. So, we are intoxicating, we are deteriorating, and our skin gets older faster due to sugar and artificial colors.

G.: Very good. Very good answer. Has anyone else had an opinion? - - M.A..

M.A.: Yes, I was laughing because I walked in with a soda bottle.

G.: Laugh.

M.A.: So, about, I think that I’ve had an argument with my girlfriend about two days ago because she tells me that I drink too much soda. And it’s true, I admit it, I drink too much soda. I can’t go one day without having a soda, or more than one.

M.: Oh, my goodness.

M.A.: Yes.

M.: Yes, because it’s a, you’ve gotten used to a drug--

M.A.: [Crosstalk] basically, I am--

FEMALE VOICE: You’re not prepared for that.

M.: It’s a drug.

M.A.: I’m addicted to soda, to colored juices that have lots of sugar. So--

G.: So, you said that you - - every day.

M.A.: Every day. And I get, I get very anxious if I don’t have it. So--

FEMALE VOICE: But it’s like something that you drink it with some food, some--?

M.A.: With everything.

FEMALE VOICE: With everything.

M.A.: With everything. So, it’s, well, my doctor told me –because I also have a history of, of my brothers who also have diabetes. So, he suggested to me that if I go on with soda, well, because it’s one of the sweet drinks I drink all the time. So, I’m prone to diabetes sooner.

FEMALE VOICE: I would say it is like an addiction.

M.A.: And it’s strange that when [crosstalk] this group, when they said focus, I thought it was more like being more alert, but I didn’t know that it was about this topic [crosstalk] soda and the, I mean, sugars.

G.: Yes, E. also has something--

E.: The same happens to me. I always have to drink soda when I eat something. When I don’t have it, it’s as if the food doesn’t taste the same. [Crosstalk].

FEMALE VOICE: That’s how I used to think, even when I had surgery at –because I would never miss out [crosstalk] on my soda. I had gallbladder surgery in January. And [Background noise] I never thought that I would have that problem, because I go to the doctor for a check-up every six months. They told me that my lungs, everything was fine. [Background noise]. And from one day to the next, that same day I got home with pain. And honey, I can’t, I can’t. I go, I had to - - trauma plane, that they took me to - - when, they first thing the woman told me was, we’re going to give you morphine. Why? Because we’re going to operate on you in two hours. And I--

M.: Oh, my goodness.

FEMALE VOICE 2: What? What? She told me, and I still have something [crosstalk] [Background noise].

M.: The gallbladder.

FEMALE VOICE 2: And they had to remove it. It was inflamed and full of stuff. - -.

M.: I am curious. With the respect of the doctors here, and you. If we are, if we are, if the talk is about drinks and sugar, and I know that what they are doing is a talk to get educated [Background noise].

G.: Well, not specifically. The talk is to know your opinion.

M.: Yes, and also, to educate ourselves. Because at the same time, we have two doctors here.

G.: Uh huh.

M.: So, they are aware of what’s being said, and they know the consequences that beverages have, because of sugar.

G.: Yes.

M.: So, I’m curious about, okay, I saw water here, but I was curious about the sodas over there.

G.: Those, those were not us.

M.: Oh. I said, oh, but come here, if sugar and sodas [crosstalk], those sodas over there.

G.: That wasn’t us. They were there [crosstalk]--

M.: I take it back.

G.: Yes. That wasn’t us. It seems that the group [crosstalk] used the room before, they [crosstalk], but they’re not ours. So, then, let’s go on. So, some of you told me that they drink soda with any meal.

J.: I, I never liked soda or fruit juices, anything like that. I’ve always drank water, but my brother and my, uh, my brother’s children drink lots of soda. And there are many that drink soda who are obese.

G.: But for you, why don’t you like them?

J.: I like them, no, I don’t like them because they have many calories, and also, uh, on my father’s side, they all died of diabetes.

G.: Okay.

J.: And my dad died of diabetes too. So, uh, I don’t want to get sick with diabetes, but it’s good that I don’t like it because, you know, it doesn’t catch my attention to drink them. I drink water and sometimes, I drink milk.

G.: Okay. So, let’s move on now. So, concerns about sugary drinks. [Background noise]. So, health professionals, like Dr. Salcedo, Dr. Chris, in the city, - - in the United States, they are worried for all the sugar contained in sodas and other sugary drinks like juices, - - Gatorade, energy drinks too. So, now, do you think that people should drink less of those drinks? I think that some already gave their opinions. Do you have other opinions about, do you think that?--

F: Well, a brother of mine, the youngest one, he doesn’t want to drink water. When he doesn’t have, he gets Kool-Aid, which comes with sugar, and he adds more sugar, but [crosstalk], and he doesn’t want to accept it. And he drinks these huge soda bottles. He can’t eat without soda.

G.: Oh, wow. So, what –another question. What do you think if you tell people that they can’t have sugary drinks, such as they forbid smoking or drinking in some places.

M.: Many that one refers to them, and they say no. Oh, it’s not your business. Leave me alone, whatever, it’s me who will die. So… Oh, no, honey, don’t but in. This is not your life; it’s me. That’s what they say when you give them some advise--

G.: [Crosstalk] opinion as if you were, like, as a rule, saying that people shouldn’t drink sugary drinks. What do you think about that?

M.: That if there were, if –because there are. But we ignore them. That--

G.: If they enforce them so much as they enforce the non-smoking, - - not drinking.

M.: Well, honey, there should be a center where that person should go for rehabilitation. Because, as there are rehabilitation centers for drugs, for smoking, for alcoholism, anonymous alcoholics, etc. But if they don’t bring us, we don’t go, you know what I mean? We don’t get educated, as I –it’s part of education, educating.

G.: Okay. First, - -.

M.A.: No, uh, I think that this would be a benefit, if the government, if the restrictions, because –just as they tried to limit large soda or juice glasses at the restaurants… But [crosstalk] now, then. But yes, if the government, let’s say, or us, the public, agree and, and we state that situation, well, perhaps, it would be beneficial for all of us.

G.: Okay. J..

J.: The biggest problem that I find that, like, when you give an opinion to someone about, oh, no, don’t drink that, drink water, you can get diabetes. Oh, it doesn’t run in my family. Oh, why did they invent them? Why did they come up with soda if one - - drink it? And sometimes, it’s the truth, because if the soda is diet, let’s say, it has few calories, it has little sugar, the die, like the die that has sodas, whatever, is harmful. So, I think that - - that would be beneficial for everyone, and likewise, they have a group and everything, and I think it’s just an addiction.

It’s possible, but at the same time, I think that if they take them away, like cigarettes, even today, there are 13-14-year old kids that look for adults, that I see that are ignorant, to buy cigarettes for that person. So, I think that even if one tries to fight and play judge, like looking for help to, oh, drop sodas, drop juices, what I think they have to focus more is on cigarettes, marihuana, because that’s also harmful. Even if many people don’t want to accept it. But the only thing about marihuana, that everyone thinks about the benefits, they say, oh, that’s helpful for epilepsy, oh, that –and sodas, what’s their benefit? And that’s what not--

M.: It has benefits, what do you mean no? Getting you to be a diabetic. Laugh.

J.: No, hard, not a benefit because it kills you.

G.: Okay. Any other opinions?

J.: I say, I don’t know if it’s possible, but I was thinking, if this were a soda, putting up a sign saying this causes [crosstalk] this and that.

J.: But look, honey, it says for cigarettes and alcohol. There is a sign on the beer bottle that says, alcohol is harmful for your health. But we keep consuming it. And they keep on manufacturing it. So, why do it? But it’s as they say in my country, a saying, that all funeral homes got together and in a talk, one of the owners said, I don’t want anybody to die, but I want my business to prosper. So, it’s the same thing. The companies that make these drinks know that they’re doing bad, but anyway, they want their business to prosper, they want it to go up. [Crosstalk].

M.A.: I agree with what she said, but, uh, as always, it’s education. It always begin, according to what we know from our culture, education begins at home. I wasn’t taught that soda was bad. I was taught that soda was good - - it’s always been. So, I think that--

G.: [Crosstalk] education.

M.A.: Since the government, well, as she says, they take advantage of more producing, more profit, more sells, but, uh, as with cigarettes, whatever, what the young woman said, we are going to have to get to that point [Background noise] that education about sugary drinks will have to begin at home.

G.: Uh huh.

M.A.: That’s what we have to get to. Because the first time that I hear a group about this.

G.: Uh huh.

M.A.: First time in my life.

G.: Because it’s something that’s being discussed more now.

M.A.: And it’s something that I’d never, never thought that we’d get to this point. [Background noise]. I think that we’ve gotten to this point that we’re consuming too much. And for years, in my case, for years.

G.: For years.

M.A.: It’s not now that I began; no.

G.: Okay. Any last opinions about… Before we go on? We’re almost, very little to finish. So, now, I’m going to share some of the messages that could be used for people to drink less soda. So, later, I’d like to ask your opinion about this. So, first, I’m going to read a paragraph to you [crosstalk] and then, - -. [Background noise]. [Background conversation]. Okay. Well, I’m going to read the messages. It’s four different messages, and then, we’ll watch the videos.

M.: Okay.

G.: So, the first message is: brain function, education and success. So, it says, [crosstalk] sodas and energy drinks contain more sugar than what someone must consume per day. There is a recent scientific research that indicates that sugar can [crosstalk] brain damage. Sodas and sugary drinks could be affecting our children’s ability to do well in school, their education and future success in life. So, just keep that in mind. The first message. [Background noise]. The second one says: illnesses, oral health and diabetes. Scientific research indicates that sugary drinks contribute to diabetes, cancer and heart disease.

In fact, a recent study showed that in the United States, sugary drinks were related to the deaths of 25,000 people from diabetes and other obesity-related diseases. Diabetes can stay with you throughout your life, and such as other diseases related to sugary drinks. One of the best things you can do for your health is to stop drinking sugary drinks. Second. Third message says: weight gain and calories. Sodas have more sugar than people think. [Background noise]. Sodas add more pounds than you think. People who drink sugar through liquids don’t feel as full as when they eat solid foods and thus, they gain more weight.

A 20 ounce soda contains more sugar than what you should eat per day. Drinking fewer sodas and sugary drinks is one of the easiest ways to lose weight. [Background noise]. Last message: advertising and marketing. Research shows that soda companies spend more money targeting Afro-American and Hispanic children than the general population. Besides television ads, several Afro-American and Hispanic people have more advertising in the street and at bus stops than in white neighborhoods. Afro-American and Hispanic children are more prone to have caries than white children. These companies target Afro-American and Hispanic children, and it’s time we do something about it.

So, those are the four messages. So, we want you to think what message would be more effective that you think would stop someone to drink fewer sodas or sugary drinks? - -. Okay. So, and we were going to show it here, but we’re having some difficulties. So, he’s going to –if you want to, I don’t know. [Background conversation]. Can you see well and all from there? [Background conversation]. Okay. Or if you could get together a bit more. I know it’s a bit uncomfortable. Like here. [Background conversation]. So, this first video is for brain function. So [Background conversation]. So, the video is in English, but I will explain. It shows two mice. So, the first mouse, when you watch, you will see that it was not given soda to drink. The second mouse they show, it was given soda to drink, and you will see that--

M.: The evolution of both, the one that drank and the one that did not.

G.: Exactly. [Background conversation]. [Background noise]. [Background conversation]. [Video playing]. So, it’s the first mouse. You saw how fast he got to where he had to [crosstalk] get. This is the one that got soda.

F: Look at what - - missing. [Background conversation].

G.: So, you saw that it doesn’t know how [crosstalk].

M.: Poor thing.

G.: So, it showed that the first one was normal. The second one had brain dysfunction.

F: Well, - - that like that.

M.: Yes, because that alters everything.

F: - -.

G.: [Background conversation]. - -. So, this one talks about the, like, diabetes and the problems it causes. [Video playing]. So, it says that if one drinks that all day, coffee, soda, - - that causes [Background noise] diabetes. [Background conversation]. So, it says there that instead of soda--

M.: Water.

G.: Water. Coffee, also, it depends on the sugar you put on, it can cause diabetes. So, instead of that, we should drink tea with no sugar, water…

M.: And even coffee without sugar.
